# Hepatocyte-specific HDAC3 ablation promotes hepatocellular carcinoma in females by suppressing Foxa1/2

**DOI:** 10.1186/s12885-023-11393-1

**Published:** 2023-09-26

**Authors:** Yahong Xu, Yongjie Zhu, Zhenru Wu, Shengfu Li, Mingyang Shao, Qing Tao, Qing Xu, Yuwei Chen, Yuke Shu, Menglin Chen, Yongjie Zhou, Yujun Shi

**Affiliations:** 1 Department of Pathology & Institute of Clinical Pathology, West China Hospital, Sichuan University, Chengdu, 610041 China; 2 Key Laboratory of Transplant Engineering and Immunology, West China Hospital, NHC, Sichuan University, Chengdu, 610041 China; 3 Department of Targeting Therapy & Immunology and Laboratory of Animal Tumor Models, Cancer Center, West China Hospital, Sichuan University, Chengdu, 610041 China; 4 Laboratory of Liver Transplantation, West China Hospital, Sichuan University, Chengdu, 610041 China

**Keywords:** HDAC3, HCC, Sex difference, IL-6, Foxa1, Foxa2, Oestrogen receptor

## Abstract

**Background:**

Hepatocellular carcinoma (HCC), the most common primary liver cancer, prevails mainly in males and has long been attributed to androgens and higher circumstantial levels of interleukin-6 (IL-6) produced by resident hepatic macrophages.

**Methods:**

Constitutively hepatocyte-specific histone deacetylase 3 (HDAC3)-deficient (*HDAC3*^*LCKO*^) mice and constitutively hepatocyte-specific HDAC3 knockout and systemic IL-6 simultaneously ablated (*HDAC3*^*LCKO*^*& IL-6*^*−/−*^) mice were used in our study to explore the causes of sex differences in HCC. Additionally, we performed human HCC tissues with an IHC score. Correlation analysis and linear regression plots were constructed to reveal the association between HDAC3 and its candidate genes. To further elucidate that HDAC3 controls the expression of Foxa1/2, we knocked down HDAC3 in HUH7 liver cancer cells.

**Results:**

We observed a contrary sex disparity, with an earlier onset and higher incidence of HCC in female mice when HDAC3 was selectively ablated in the liver. Loss of HDAC3 led to constant liver injury and the spontaneous development of HCC. Unlike the significant elevation of IL-6 in male mice at a very early age, female mice exhibit stable IL-6 levels, and IL-6 ablation did not eliminate the sex disparity in hepatocarcinogenesis in HDAC3-deficient mice. Oestrogen often protects the liver when combined with oestrogen receptor alpha (ERα); however, ovariectomy in HDAC3-ablated female mice significantly delayed tumourigenesis. The oestrogen-ERα axis can also play a role in tumour promotion in the absence of Foxa1 and Foxa2 in the receptor complex. Loss of HDAC3 profoundly reduced the expression of both Foxa1 and Foxa2 and impaired the binding between Foxa1/2 and ERα. Furthermore, a more frequent HDAC3 decrease accompanied by the simultaneous Foxa1/2 decline was found in female HCC compared to that in male HCC.

**Conclusion:**

In summary, we reported that loss of HDAC3 reduces Foxa1/2 and thus promotes HCC development in females in an oestrogen-dependent manner.

**Supplementary Information:**

The online version contains supplementary material available at 10.1186/s12885-023-11393-1.

## Background

Primary liver cancer (PLC), including hepatocellular carcinoma (HCC), which comprises 75-85% of cases, was the sixth most commonly diagnosed cancer and the third leading cause of cancer death worldwide in 2020 [1]. Chronic infections with hepatitis B virus (HBV) or hepatitis C virus (HCV), aflatoxin-contaminated foods, heavy alcohol intake, excess body weight, type 2 diabetes, and smoking have been identified as key risk factors for HCC [2]. HCC has a striking predominance in males [3–5]. According to 2020 estimates from GLOBOCAN, the estimated number of cases for men is 632,320, more than twice that of women, with an age-standardized rate (ASR) of 14.1 [1]. This sex difference in HCC has been well explained by sex hormones [6].

Diverse sex hormones serve different roles in animal research, with androgen serving as a tumour promoter and oestrogen working as a tumour inhibitor [7–9]. Oestrogen has also been discovered to slow the progression of HCC by controlling tumour cell invasion, proliferation, and apoptosis by lowering the expression of matrix metallopeptidase 2 (MMP2), matrix metallopeptidase 9 (MMP9), proliferating cell nuclear antigen (PCNA), cyclin A, cyclin D1, and B-cell lymphoma-2 (Bcl-2) [10]. Furthermore, an increased incidence of HCC is observed in female mice lacking oestrogen receptor alpha (ERα), whereas male mice lacking androgen receptor (AR) acquire resistance to HCC [11, 12]. HCC growth can also be slowed by ERα-mediated activation of protein tyrosine phosphatase receptor type O (PTPRO), a tumour inhibitor in many malignancies [13]. These data suggest that the oestrogen signalling axis protects women from developing HCC.

Class I HDAC members include HDAC 1, 2, 3, and 8 [14]. Among HDACs, HDAC3 plays an extraordinary role in DNA damage control [15], but the signalling cascade in HCC is far from clear. Using individual class I HDAC member-deficient mice, we have demonstrated that K9 in histone H3 (H3K9), which is the critical site for the assembly of DNA damage response (DDR) complexes, is exclusively targeted by HDAC3 [16]. Ablation of HDAC3 disrupts the deacetylation of H3K9ac and the consequent trimethylation of H3K9 (H3K9me3) and impairs the assembly of the DDR complex, leading to the accumulation of damaged DNA and ultimately spontaneous HCC.

During the breeding of liver-conditional HDAC3-ablation mice (*HDAC3*^*LCKO*^), we noticed that female mice not only had spontaneous HCC significantly earlier than males but also had a higher incidence. It has been reported that oestrogen-mediated inhibition of interleukin-6 (IL-6) reduces the risk of liver cancer in women [17]. Although it seems to be contrary to the phenotype, we found a regulatory relationship between the IL-6 signalling pathway and sex hormones in HCC. Considering that we have found that HDAC3 could promote the proliferation of HCC cells by promoting IL-6-STAT3 signalling [18], the sex-specific effects of HDAC3 deficiency might be related to IL-6. Based on previous studies, Foxa1 and Foxa2, members of the forkhead (Foxa) family of transcription factors, are often associated with sex hormones [19]. Both Foxa1 and Foxa2 promote prostate cancer cell growth through the androgen-mediated signalling pathway [20]. In breast cancer, Foxa1 promotes breast cancer cell growth through the oestrogen-ERα signalling axis [21]. Although the sexual dimorphism of HCC was reversed in Foxa1/2-deficient mice [22], the role of Foxa1/2 in HCC remains unclear. Importantly, only in breast cancer has it been indicated that suppression of HDAC3 expression can downregulate ERα, a protective factor in breast cancer, but this has not been reported in liver cancer [23, 24].

In this study, we found that HDAC3 deficiency causes sex differences in HCC and that females develop HCC much earlier than males. Mechanistically, loss of HDAC3 suppresses the expression of Foxa1 and Foxa2, which might release the carcinogenic effect of oestrogen. Our findings will help understand the etiology of HCC in women and provide a molecular framework that should be useful for the design of new therapeutic strategies.

## Materials and methods

### Human HCC samples

For the retrospective cohort study, human HCC tissues resected from 341 diagnosed patients with complete clinical information were obtained from the West China Hospital of Sichuan University between 2009 and 2015. According to the signal distribution and intensity, the expression levels of HDAC3, Foxa1, and Foxa2 were scored by three pathologists using a blinded method. Briefly, at least five 400× magnified areas were examined and scored for signal distribution as follows: 0, < 5% stained; 1, 5–25% stained; 2, 25–75% stained; and 3, > 75% stained. The intensity of staining was scored as follows: 1, weak; 2, moderate; and 3, intense. Tissues with an IHC score (product of signal distribution score and staining intensity score) of 0–3 were designated tissues with low expression, and those with scores of 4–9 were designated tissues with high expression. All methods were carried out in accordance with relevant guidelines and regulations. All patient materials were obtained with written informed consent. The procedures used for human sample collection and use were approved by the ethics committee of West China Hospital, Sichuan University (Chengdu, China). The information of patients is listed in Supplementary Table S1. To reveal the association between HDAC3 and its candidate genes, correlation analysis was performed, and linear regression plots were constructed using GraphPad Prism 8.

### Animals

Frozen *HDAC3*^*loxP/loxP*^ embryos were purchased from the European Mouse Mutant Archive (EMMA). Alb-Cre and systemic IL-6 knockout (*IL-6*^*−/−*^) mice were purchased from Shanghai Bio Model Organism Science & Technology Development Co., Ltd, China. *HDAC3*^*loxP/loxP*^ mice were intercrossed with Albumin-Cre transgenic mice to obtain constitutively hepatocyte-specific HDAC3-deficient (*HDAC3*^*LCKO*^) mice as previously described [16, 18]. *HDAC3*^*LCKO*^ mice were intercrossed with *IL-6*^*−/−*^ mice to obtain constitutively hepatocyte-specific HDAC3 knockout and systemic IL-6 simultaneously ablated (*HDAC3*^*LCKO*^&*IL-6*^*−/−*^) mice. In addition, we performed ovariectomy (OVX) on *HDAC3*^*LCKO*^ female mice at one month of age, as described previously [25]. All mice were fed a standard chow diet and housed on corncob bedding under SPF conditions. All experiments were performed in accordance with relevant guidelines and regulations. The animal procedures and care were conducted following national and international laws and policies and approved by the Animal Care and Use Committee of Sichuan University.

### H&E staining and immunohistochemical staining

Mouse liver tissues harvested at the indicated points were fixed in 10% buffered formalin for 48 h followed by dehydration in ethyl alcohol and then embedded in the optimal cutting temperature (OCT) compound, and 4 μm thick sections were prepared by manual rotary sectioning for H&E staining and immunohistochemical staining. The antibodies used in this study are listed in Supplementary Table S2.

### Cell culture and siRNA transfection

HCC cell lines were obtained from the American Type Culture Collection (ATCC), and each cell authentication was also performed by ATCC using short tandem repeat (STR) markers. Mycoplasma testing was performed using a MycoAlert™ Mycoplasma Detection Kit (Basel, Switzerland) according to the manufacturer’s recommended protocols. The cells were maintained in Dulbecco’s modified Eagle’s medium (DMEM) supplemented with 10% fetal bovine serum, 2 mM glutamine, and 100 U/ml penicillin/streptomycin. All cells were maintained at 37°C in a 5% (v/v) CO_2_ atmosphere and subcultured every 3 days. Transfection with siRNA against the HDAC3 gene (siHDAC3: 5’-CCGCCAGACAAUCUUUGAAdTdT-3’) was performed using Lipofectamine® 3000 [19]. Scrambled siRNA was used as a control. The effect of siRNAs was confirmed by Western blot analysis.

### Western blotting

Liver tissue was lysed in RIPA buffer and centrifuged at 13,000 rpm and 4 °C for 15 min. The samples were subjected to protein electrophoresis, the membrane was transferred and closed, the primary antibody was incubated overnight, and then the membrane was incubated with the secondary antibody. The developing working solution was configured, and the membrane was transferred to the gel imaging system for exposure and photography. Finally, quantitative analysis was performed using ImageJ software. The antibodies used in this study are listed in Supplementary Table S2. All full-length blots are presented in Supplementary materials.

### Real-time RT-PCR analysis

RNA from tissues was extracted using TRIzol reagent (Invitrogen, California, USA) and purified using the RNeasy kit (Qiagen, California, USA). cDNA was generated using the iScript cDNA synthesis kit (Bio-Rad, California, USA), and qRT-PCR was performed with the Bio-Rad CFX96 System. The primer sequences of each gene are available when needed.

### Enzyme-linked immunosorbent assay

Mouse serum was collected at the indicated times. An ELISA kit was purchased from Elabscience Biotechnology, Wuhan, China. The assay was carried out according to the manufacturer’s instructions. Absorbance values were measured at 450 nm using an enzyme marker (Thermo, Massachusetts, USA) and finally analyzed and calculated.

### Immunoprecipitation

Liver tissue lysates were prepared using RIPA buffer containing a mixture of phosphatase inhibitors and protease inhibitors. A reversible immunoprecipitation System Kit (Millipore, Massachusetts, USA) was used according to the manufacturer’s instructions.

### Statistical analysis

All experimental data represent the results of more than three experiments and are presented as the mean ± standard deviation. The data were analyzed by SPSS statistical software version 19.0 (Massachusetts, USA) and were processed by one-way ANOVA. A P-value less than 0.05 was considered significant. * *p* ＜ 0.05, ** *p* ＜ 0.01, *** *p* ＜ 0.001, and **** *p* ＜ 0.0001.

## Results

### Hepatocyte-specific deletion of HDAC3 results in earlier onset of HCC in female mice

**Fig. 1:**
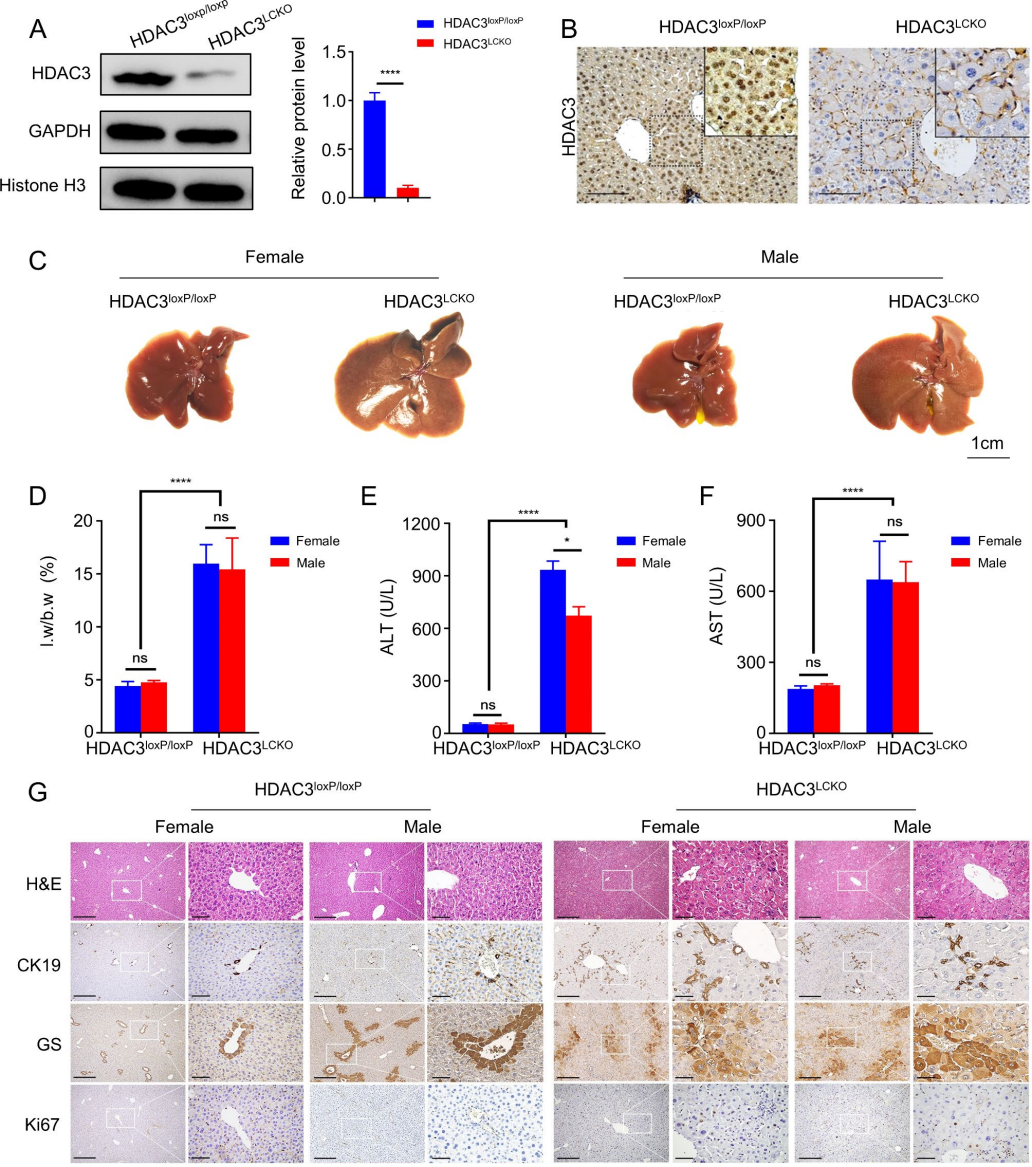
Significant hepatocyte damage in 2-month-old *HDAC3*^*LCKO*^ mice. **(A)** Western blot detection of HDAC3 protein in livers from *HDAC3*^*LCKO*^ mice. GAPDH and histone H3 were used as the loading controls. **(B)** Immunohistochemistry staining shows the negative expression of HDAC3 in *HDAC3*^*LCKO*^ hepatocytes. Scale bar, 100 μm. **(C)** Gross morphology of *HDAC3*^*loxP/loxP*^ and *HDAC3*^*LCKO*^ livers. **(D)** The ratio of liver weight (l.w) to body weight (b.w) between *HDAC3*^*loxP/loxP*^ mice and *HDAC3*^*LCKO*^ mice. **(E-F)** Serum levels of ALT and AST. **(G)** H&E staining and immunohistochemistry staining of glutamine synthetase (GS), cytokeratin 19 (CK19), and Ki67 in livers from 2-month-old *HDAC3*^*LCKO*^ mice. Scale bar, 100 μm. * *p* ＜ 0.05, **** *p* ＜ 0.0001, and ns, not significant

**Fig. 2:**
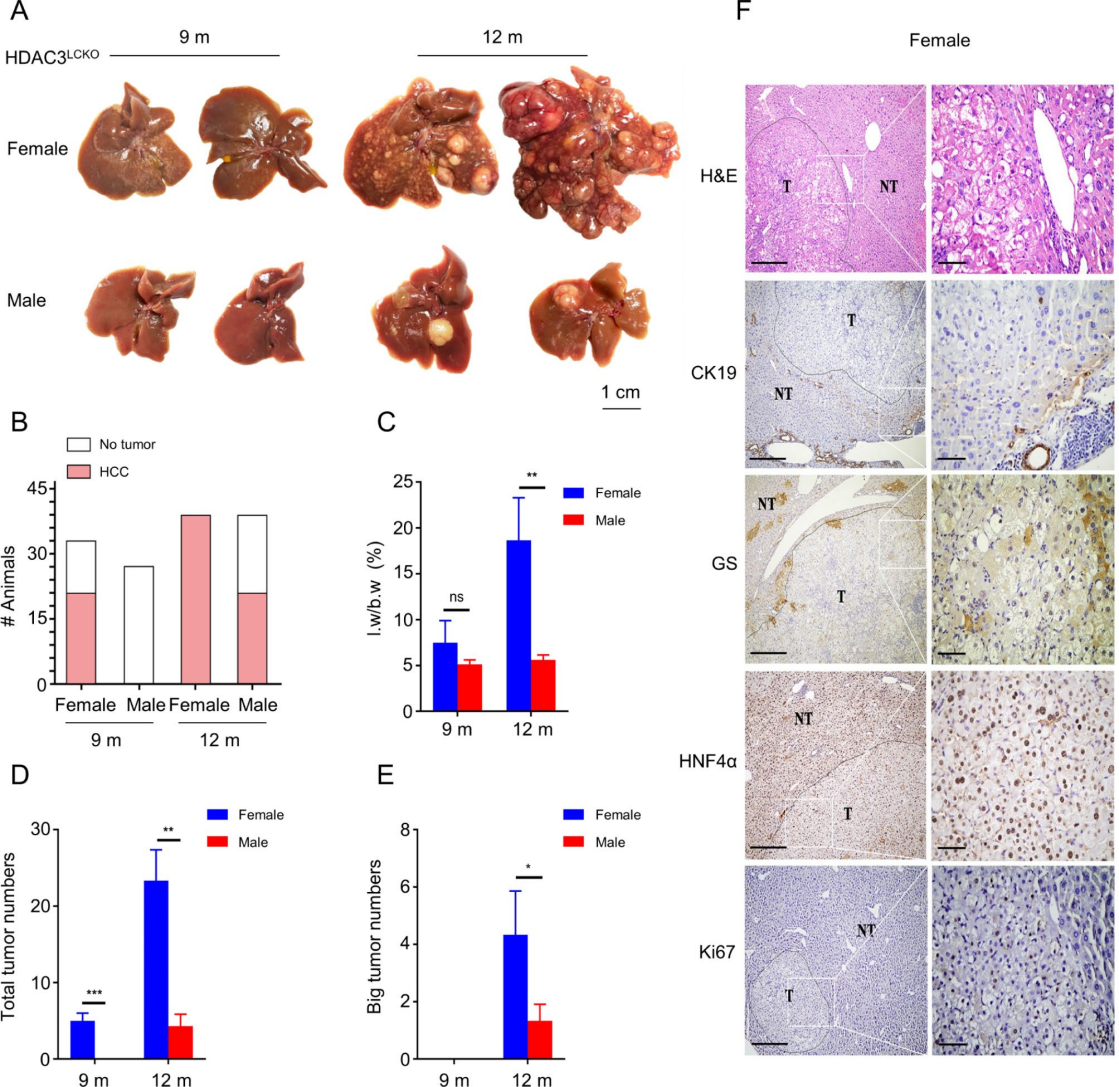
HDAC3 deletion results in earlier HCC onset in female mice. **(A)** Gross morphology of *HDAC3*^*LCKO*^ livers at 9 and 12 months old. **(B)** The number of female *HDAC3*^*LCKO*^ mice and male *HDAC3*^*LCKO*^ mice with liver nodules. **(C)** The ratio of liver weight (l.w) to body weight (b.w) in *HDAC3*^*LCKO*^ mice at 9 and 12 months old. **(D)** The number of tumours in female and male *HDAC3*^*LCKO*^ mice at the indicated times. **(E)** The number of tumours larger than 5 mm in diameter in female and male *HDAC3*^*LCKO*^ mice. **(F)** Histological detection of the livers of 9-month-old female *HDAC3*^*LCKO*^ mice. T, tumour; NT, not tumour. Scale bar, 100 μm. * *p* ＜ 0.05, ** *p* ＜ 0.01, *** *p* ＜ 0.001, and ns, not significant

As previously stated, we created a hepatocyte-specific HDAC3-ablation mouse (*HDAC3*^*LCKO*^) [16, 18]. HDAC3 was entirely deleted in the hepatocytes of *HDAC3*^*LCKO*^ mice, according to Western blot analysis and immunohistochemistry (Fig. 1A and B). At 2 months of age, *HDAC3*^*LCKO*^ mice had significantly larger livers (Fig. 1 C and 1D) and higher serum ALT and AST levels (Fig. 1E and F). ALT levels in *HDAC3*^*LCKO*^ females were substantially higher than those in *HDAC3*^*LCKO*^ males. Meanwhile, staining for Ki67, a proliferative cell marker, showed a large abundance of proliferating cells in the mutant liver (Fig. 1G), indicating compensatory regeneration upon spontaneous liver injury. *HDAC3*^*LCKO*^ mice had substantial expansion and steatosis of hepatocytes in histology, as revealed by H&E staining (Fig. 1G). Glutamine synthetase (GS) is specifically expressed in the central venous region of the hepatic lobule. However, GS^+^ cells were irregularly scattered in the lobules of *HDAC3*^*LCKO*^ livers, showing disarrangement of the lobules. Cytokeratin 19 (CK19) is a marker for bile duct cells and a subset of hepatic progenitor cells (HPCs). Active HPC expansion, known as a ductular reaction, is often considered to repair the chronically injured liver by differentiating into hepatocytes or bile duct cells. The livers of *HDAC3*^*LCKO*^ mice showed substantial proliferation of CK19^+^ cells (Fig. 1G), indicating persistent damage to the mutant liver. Notably, at 9 months of age, liver nodules were observed in more than half of the *HDAC3*^*LCKO*^ female mice (64%, 21/33), but not in the *HDAC3*^*LCKO*^ male mice (0%, 0/27) (Fig. 2A and B). The liver-to-body weight ratio in females was not significantly different from that in males (Fig. 2C). The average number of liver nodules per female mouse was 5, and almost all tumours were smaller than 5 mm (Fig. 2D and E). When stained with H&E, tumour cells had basophilic cytoplasm and large, irregular nuclei (Fig. 2F). Hepatocyte nuclear factor 4α (HNF4α) is a marker for the unique recognition of hepatocytes. HNF4α^+^ cells were found throughout the tumour, while CK19^+^ cells were found outside the tumour (Fig. 2F), indicating that the tumour originated from hepatocytes. In addition, Ki67 immunohistochemistry staining indicated the active growth of the tumour cells (Fig. 2F). All *HDAC3*^*LCKO*^ female mice developed large liver tumours (> 5 mm) at 12 months. Although tumour nodules were seen in the livers of *HDAC3*^*LCKO*^ male mice, the number of total tumours and nodules larger than 5 mm were much lower in males (Fig. 2A, B and D, and E). As a result, the liver-to-body weight ratio in females was much higher than that in males (Fig. 2C). Taken together, *HDAC3*^*LCKO*^ female mice developed spontaneous HCC much earlier than male mice.

### Serum IL-6 levels are stable in *HDAC3*^*LCKO*^ females before HCC development

**Fig. 3:**
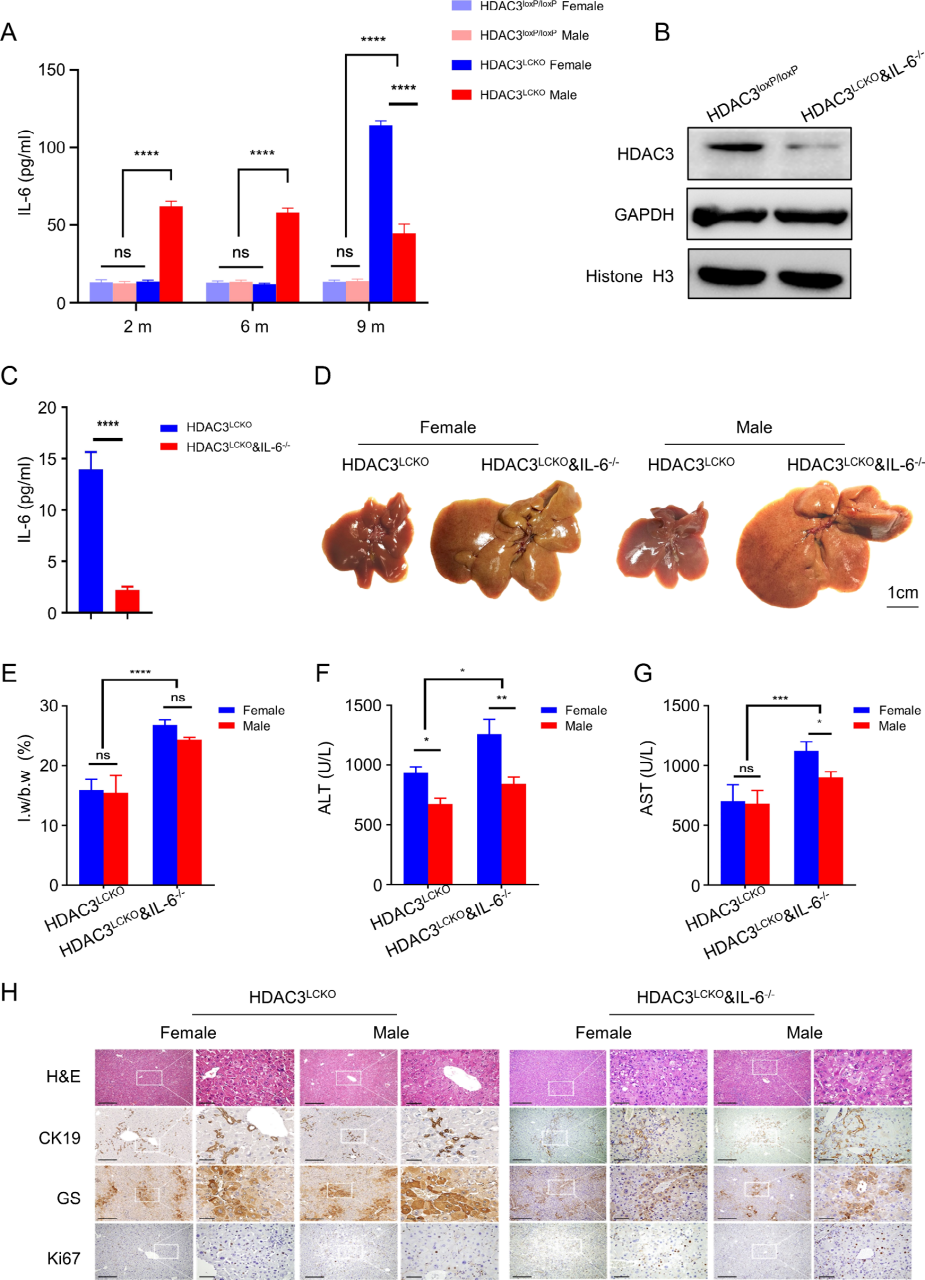
HDAC3 deletion resulted in higher IL-6 levels in female mice and more severe liver damage was observed in *HDAC3*^*LCKO*^&*IL-6*^*−/−*^ mice at 2 months of age. **(A)** Serum IL-6 levels in mice of different genotypes and sexes at 2, 6, and 9 months. **(B)** Western blot detection of HDAC3 protein in the liver. **(C)** Serum IL-6 levels in *HDAC3*^*LCKO*^ and *HDAC3*^*LCKO*^&*IL-6*^*−/−*^ mice. **(D)** Gross morphology of *HDAC3*^*LCKO*^ and *HDAC3*^*LCKO*^&*IL-6*^*−/−*^ livers. **(E)** The ratio of liver weight (l.w) to body weight (b.w) between *HDAC3*^*LCKO*^ and *HDAC3*^*LCKO*^&*IL-6*^*−/−*^ mice. **(F-G)** Serum AST and ALT levels. **(H)** H&E staining and immunohistochemistry staining of GS, CK19, and Ki67 in livers from 2-month-old *HDAC3*^*LCKO*^ mice. Scale bar, 100 μm. * *p* ＜ 0.05, ** *p* ＜ 0.01, *** *p* ＜ 0.001, **** *p*＜ 0.0001, and ns, not significant

IL-6 has a crucial role in promoting HCC. By binding to the soluble receptor sIL-6R, IL-6 activates the JAK-STAT3 signalling pathway to promote tumour proliferation [26]. Importantly, higher IL-6 levels increase the risk of HCC in males [27]. While IL-6 ablation eliminates the sex difference in hepatocarcinogenesis [17], demonstrating that IL-6 is involved in sex differences in HCC in a unique way. We recently reported that HDAC3 promotes liver regeneration and boosts HCC cell proliferation by increasing IL-6-STAT3 activation [18], which suggested that IL-6 signalling might be aberrantly activated in female mice when HDAC3 is selectively deleted in the liver. Strikingly, despite having much higher ALT levels than males at 2 and 6 months, IL-6 in *HDAC3*^*LCKO*^ females remained normal and was significantly lower than that in males (Fig. 3A). Only when *HDAC3*^*LCKO*^ females developed HCC at nine months of age were IL-6 levels noticeably elevated, even exceeding male levels (Fig. 3A). Overall, *HDAC3*^*LCKO*^ females had lower IL-6 levels but earlier HCC onset, which suggested that the sex disparity could not be uniquely attributed to the IL-6 difference.

### IL-6 deprivation does not erase the sex difference in *HDAC3*^*LCKO*^mice

**Fig. 4:**
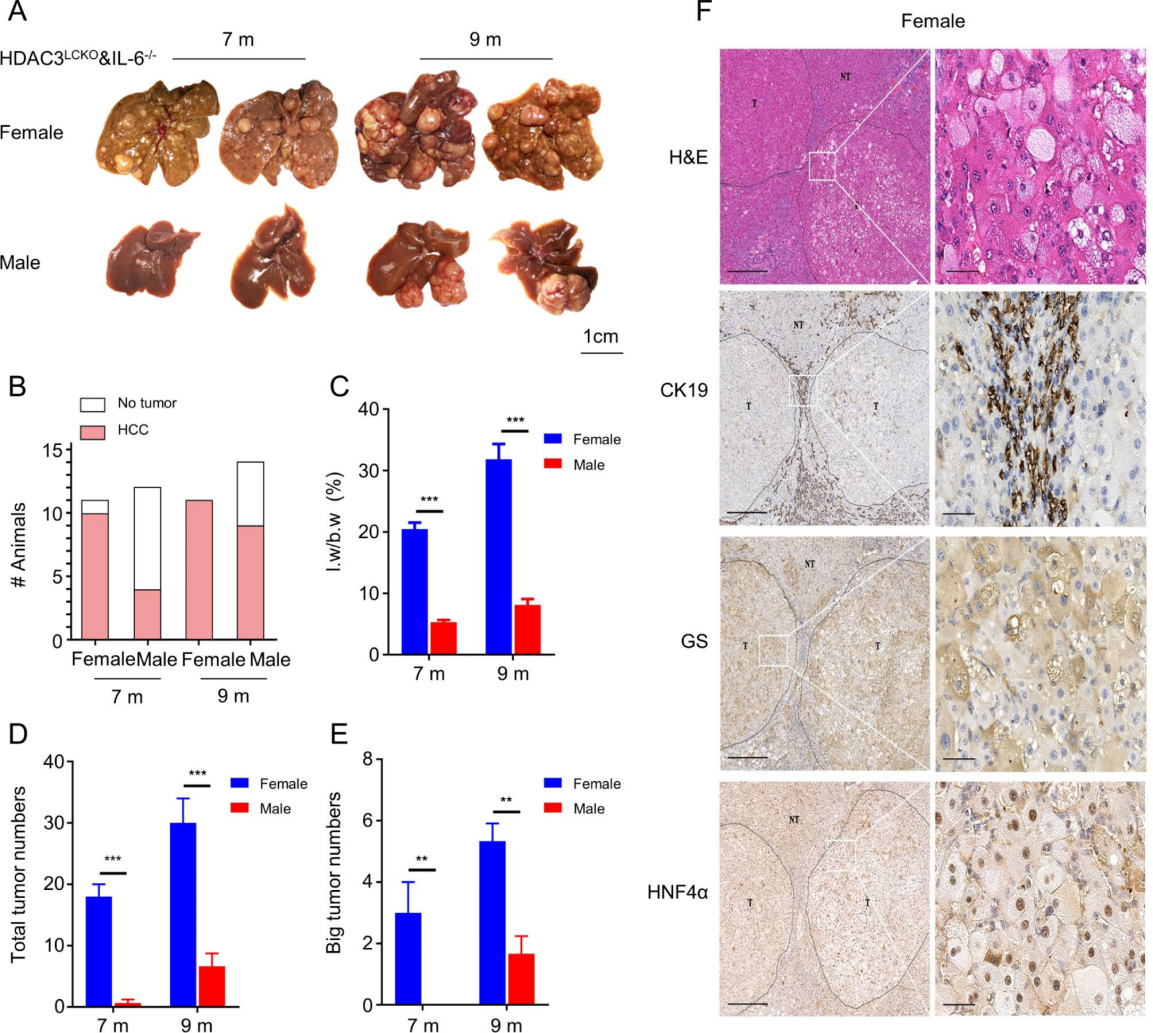
*HDAC3*^*LCKO*^&*IL-6*^*-/-*^ female mice developed HCC earlier than males. **(A)** Gross liver morphology in *HDAC3*^*LCKO*^&*IL-6*^*−/−*^ mice at 7 and 9 months old. **(B)** The number of female *HDAC3*^*LCKO*^&*IL-6*^*−/−*^ mice and male *HDAC3*^*LCKO*^&*IL-6*^*−/−*^ mice with liver nodules. **(C)** The ratio of liver weight to body weight in *HDAC3*^*LCKO*^&*IL-6*^*−/−*^ mice at 7 and 9 months old. **(D)** The number of tumours in female *HDAC3*^*LCKO*^&*IL-6*^*−/−*^ mice and male *HDAC3*^*LCKO*^&*IL-6*^*−/−*^ mice. **(E)** The number of tumours larger than 5 mm in diameter in female *HDAC3*^*LCKO*^&*IL-6*^*−/−*^ mice and male *HDAC3*^*LCKO*^&*IL-6*^*−/−*^ mice. **(F)** Histological detection of the liver in 7-month-old female *HDAC3*^*LCKO*^&*IL-6*^*−/−*^ mice. T, tumour; NT, not tumour. Scale bar, 100 μm. * *p* ＜ 0.05, ** *p* ＜ 0.01, and *** *p* ＜ 0.001

We further created *HDAC3*^*LCKO*^&*IL-6*^*−/−*^ mice to determine whether IL-6 plays a vital role in HCC in *HDAC3*^*LCKO*^ mice. Protein blotting and measurement of serum IL-6 confirmed the successful construction of double-gene knockout mice (Fig. 3B and C). *HDAC3*^*LCKO*^&*IL6*^*−/−*^ mice had an increase in liver size by 2 months old (Fig. 3D), and the liver-to-body weight ratio increased from approximately 15% in *HDAC3*^*LCKO*^ mice to 26% in *HDAC3*^*LCKO*^&I*IL-6*^*−/−*^ mice (Fig. 3E). ALT levels increased almost twofold in *HDAC3*^*LCKO*^&*IL-6*^*−/−*^ mice compared to *HDAC3*^*LCKO*^ mice (Fig. 3F and G). Although the liver-to-body weight ratio did not show a significant difference between the sexes, the ALT and AST levels were still higher in females than in males. Interestingly, the histological differences between *HDAC3*^*LCKO*^&*IL-6*^*−/−*^ males and females were not visible, as shown by H&E, CK19, GS, and Ki67 staining (Fig. 3H). Notably, by 7 months old, the livers of *HDAC3*^*LCKO*^&*IL-6*^*−/−*^ female mice had a considerable number of tumour nodules (91%, 10/11), whereas the livers of *HDAC3*^*LCKO*^&*IL-6*^*−/−*^ male mice had only smaller tumour nodules (36%, 4/11) (Fig. 4A and B). The average number of liver nodules per female and male mouse was 18 and 0.67, respectively, with 3 and 0 tumours larger than 5 mm in diameter, respectively (Fig. 4D and E). The livers of *HDAC3*^*LCKO*^&*IL-6*^*−/−*^ female mice exhibited a larger liver-to-body weight ratio (Fig. 4C). Again, the tumours were proven to be HCC by H&E staining and immunostaining for CK19, GS, and HNF4α (Fig. 4F). At 9 months of age, all *HDAC3*^*LCKO*^&*IL-6*^*−/−*^ female mice developed spontaneous HCC (100%, 11/11), while only over half of *HDAC3*^*LCKO*^&*IL-6*^*−/−*^ males developed tumours (65%, 9/14) (Fig. 4A and B). Increased tumour nodules were found in 9-month-old *HDAC3*^*LCKO*^&*IL-6*^*−/−*^ male mice, and the average number of liver tumours per *HDAC3*^*LCKO*^&*IL6*^*−/−*^ male mouse was 7, which was significantly smaller than that of the females (Fig. 4D and E). Remarkably, despite having more spontaneous HCC than *HDAC3*^*LCKO*^ males, *HDAC3*^*LCKO*^&*IL-6*^*−/−*^ males did not develop HCC earlier than *HDAC3*^*LCKO*^&*IL-6*^*−/−*^ females. Collectively, IL-6 deprivation in *HDAC3*^*LCKO*^ mice did not remove sex differences but accelerated HCC progression in both sexes and *HDAC3*^*LCKO*^&*IL-6*^*−/−*^ female mice developed spontaneous HCC approximately two months earlier than *HDAC3*^*LCKO*^ females.

### Ovariectomy prevents the early onset of HCC in HDAC3-deficient female mice

**Fig. 5:**
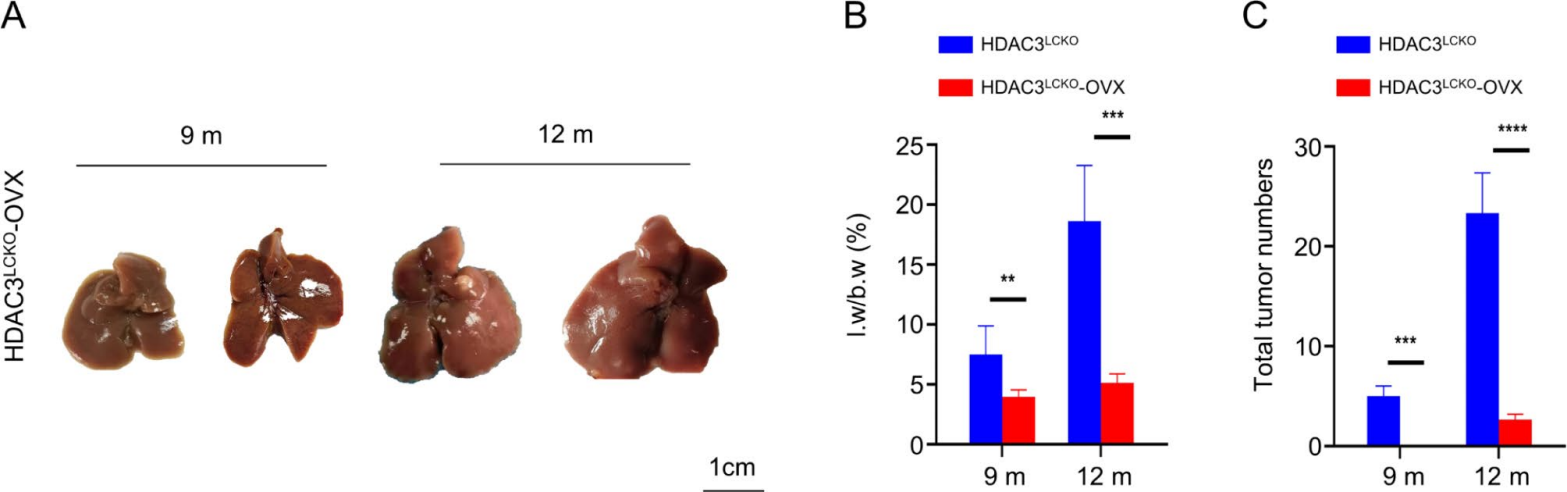
Ovariectomy significantly delayed tumorigenesis. **(A)** Gross morphology of *HDAC3*^*LCKO*^-OVX livers at 9 and 12 months old. **(B)** The number of *HDAC3*^*LCKO*^ and *HDAC3*^*LCKO*^-OVX mice with liver nodules at the indicated times. **(C)** The ratio of liver weight (l.w) to body weight (b.w) in *HDAC3*^*LCKO*^ and *HDAC3*^*LCKO*^-OVX mice. ** *p* ＜ 0.01, *** *p* ＜ 0.001, and **** *p* ＜ 0.0001

The observation that HDAC3 deficiency specifically contributed to early HCC in female mice prompted us to investigate the association between HDAC3 and oestrogen. Although ERα-mediated oestrogen signalling has been shown to inhibit hepatocarcinogenesis [9, 10], oestrogen itself has been shown to induce cancer due to the genotoxicity of its metabolites [28]. To elucidate whether the effect of HDAC3 on HCC is regulated by oestrogen, we performed ovariectomy (OVX) on *HDAC3*^*LCKO*^ female mice and examined tumour progression. To our surprise, no tumours were observed in the livers of females at nine months, and only small tumours appeared at 12 months old after OVX (Fig. 5A). The livers of *HDAC3*^*LCKO*^ -OVX mice displayed a decreased liver weight-to-body weight ratio compared with those of *HDAC3*^*LCKO*^ mice (Fig. 5B). Most intriguingly, the total number of tumours in the *HDAC3*^*LCKO*^ mice was reduced by 100% at nine months and by approximately 89% at 12 months after OVX, indicating that oestrogen functions as a tumour promoter (Fig. 5C). Our data support that oestrogen signalling promotes female HCC in the absence of HDAC3.

### Loss of HDAC3 reduces foxa1 and foxa2

**Fig. 6:**
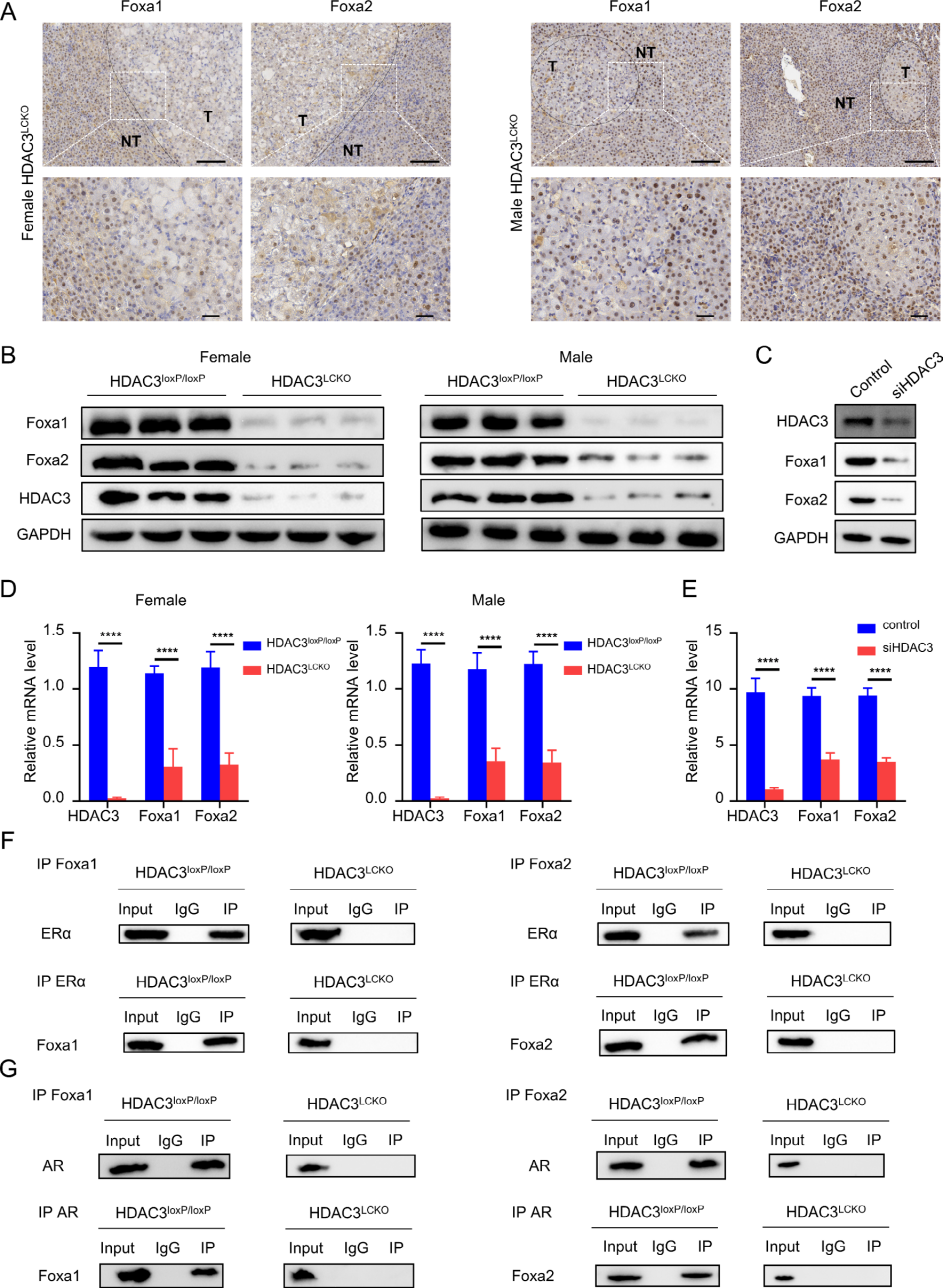
HDAC3 ablation suppresses Foxa1 and Foxa2 expression and their binding to ERα and AR. **(A)** Immunohistochemical detection of Foxa1 and Foxa2 in the livers of *HDAC3*^*LCKO*^ mice. Scale bar, 100 μm. **(B)** Western blot detection of Foxa1 and Foxa2 protein expression in the livers of *HDAC3*^*LCKO*^ mice. **(C)** Western blotting indicating that knockdown of HDAC3 reduced Foxa1 and Foxa2 in HUH7 cells. **(D)** The mRNA level of HDAC3, Foxa1, and Foxa2 in *HDCA3*^*loxP/loxP*^ and *HDAC3*^*LCKO*^ mice. **(E)** The mRNA level of Foxa1 and Foxa2 in HUH7 cells. **(F)** The interactions between ERα and Foxa1/2 were analyzed by co-immunoprecipitation (co-IP). **(G)** The interactions between AR and Foxa1/2 were analyzed by co-immunoprecipitation (co-IP).

In the livers of females, Foxa1/2 and ERα cooperate to regulate genes involved in cancer resistance pathways such as xenobiotic metabolism and detoxification, DNA biosynthesis and replication, and cell cycling and proliferation, but the coregulation disappeared in Foxa1/2-deficient mice with or without carcinogen administration [22]. Thus, oestrogen has both hepatoprotective and tumour-promoting effects, depending on ERα activity. Foxa1 and Foxa2 create a transcriptional complex with ERα to suppress HCC, but oestrogen promotes HCC with Foxa1/2 ablation, which is comparable to the behaviour of the *HDAC3*^*LCKO*^ animals in our study. This implied that HDAC3 might regulate the expression of Foxa1/2. To explore this possibility, we examined the expression of these two factors in *HDAC3*^*LCKO*^ mice. Indeed, in liver tissues, ablation of HDAC3 inhibited the expression of Foxa1 and Foxa2, according to western blot analysis and immunohistochemistry (Fig. 6A and B). To further elucidate that HDAC3 controls the expression of Foxa1/2, we knocked down HDAC3 in HUH7 liver cancer cells. As shown in Fig. 6C, the protein expression of Foxa1 and Foxa2 was subsequently decreased in HUH7 cells. Compared with *HDAC3*^*loxP/loxP*^ mice, the mRNA level of Foxa1 and Foxa2 was reduced in *HDAC3*^*LCKO*^ mice regardless of gender (Fig. 6D). Similarly, HDAC3 silence significantly decreased the mRNA level of Foxa1 and Foxa2 in HUH7 cells (Fig. 6E). These findings suggested that HDAC3 regulates Foxa1 and Foxa2 expression at the transcription level. Remarkably, the absence of ERα binding to Foxa1/2 was further confirmed by immunoprecipitation (Fig. 6F). Thus, the protective effect of the oestrogen-ERα signalling axis on HCC might have been abolished by Foxa1/2 deficiency. Foxa1/2 has been found to interact with the AR in male mouse livers [22]. We found that both Foxa1 and Foxa2 were bound to AR in the *HDAC3*^*loxP/loxP*^ male mouse livers. On the contrary, the interaction between Foxa1/2 and AR was abolished in the *HDAC3*^*LCKO*^ male mouse livers, as shown by immunoprecipitation assays (Fig. 6G), which demonstrated that Foxa1/2 is also essential for androgen signalling in promoting hepatocarcinogenesis in male mice. We next examined the expression of several Foxa1/2 target genes, including BTG1 (B-cell translocation gene 1), an anti-proliferative factor, FGL1 (fibrinogen-like 1), an acute phase reactant for inflammatory response, ABCC4 (ATP-binding cassette, sub-family C, member 4), which mediates the membrane transport of various molecules in multi-drug resistance, and PPM1L (protein phosphatase, Mg2/Mn2 dependent, 1 L), a tumour suppressor. Consistent with the previous report by Li et al. [22], we also found that PPM1L expression was reduced, but FGL1, BTG1, and ABCC4 expression were increased in *HDAC3*^*LCKO*^ livers compared to *HDAC3*^*loxP/loxP*^ liver (Figure S1). Overall, our findings demonstrated that HDAC3 might regulate Foxa1/2 expression, which correlates with HCC development in females.

### Female HCC patients display a higher percentage of low HDAC3 expression

**Fig. 7:**
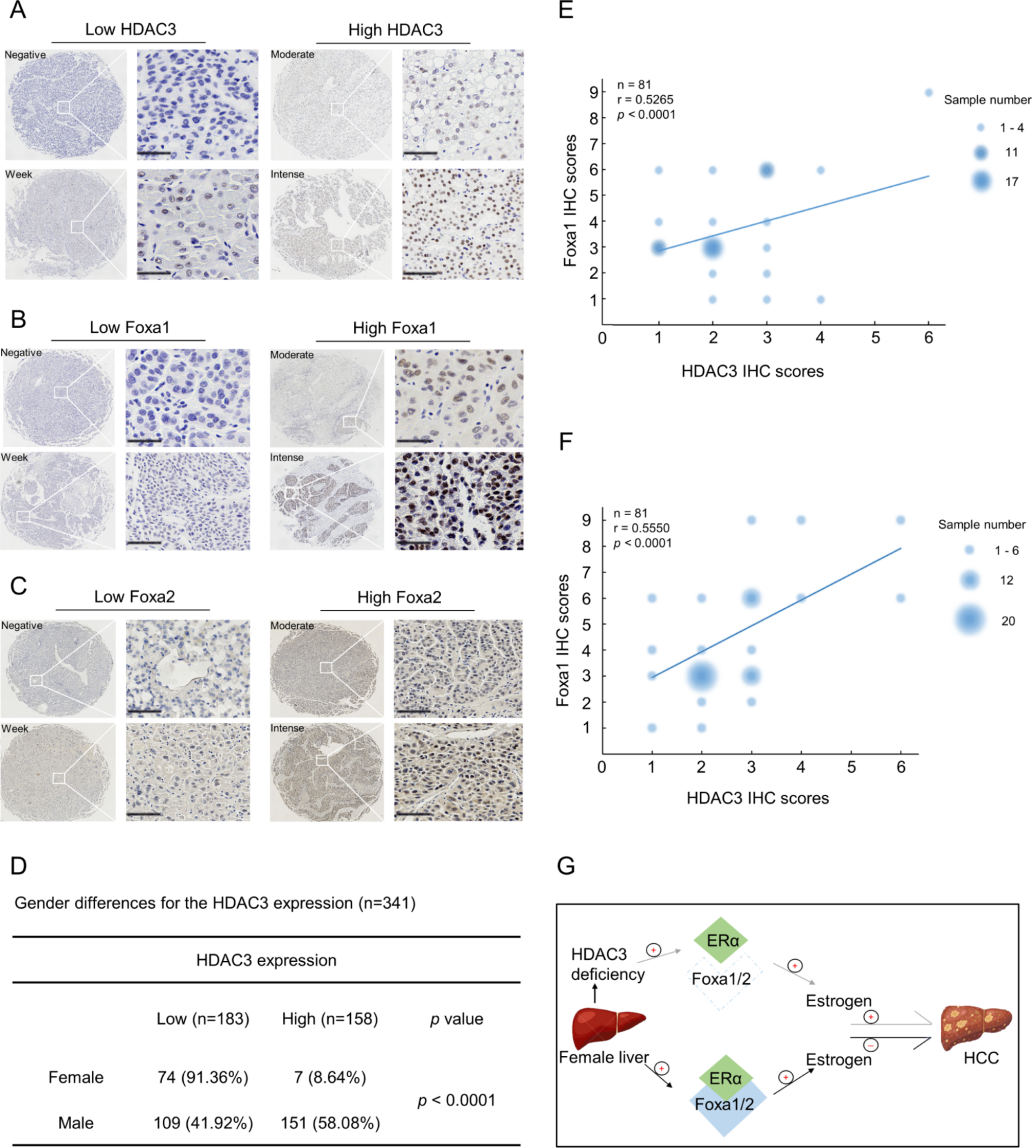
Human HCCs display sex differences in HDAC3 levels. **(A)** staining intensity of HDAC3 in HCC. Scale bar, 100 μm. **(B-C)** Staining intensity of Foxa1 and Foxa2 in HCC. Scale bar, 100 μm. **(D)** The chi-square test revealed HDAC3 expression was presumably lower in female HCC. **(E-F)** Correlation analysis revealed that Foxa1 (r = 0.5265; p < 0.0001) and Foxa2 (r = 0.5550; p < 0.0001) were positively associated with HDAC3 expression in female HCC patients. **(G)** Diagram of the hypothetical roles of HDAC3 in female HCC development by regulating the Foxa1/2 signalling pathway. * *p* ＜ 0.05 and **** *p* ＜ 0.0001

We further evaluated whether female HCC patients have lower HDAC3 and Foxa1/2 expression since oestrogen can prevent HCC development in women if Foxa1/2 is sufficient. Scoring of the immunohistochemistry staining of human HCC tissues grouped 341 patients into the high HDAC3 expression group (HDAC3^high^, score 4–9) and the low HDAC3 expression group (HDAC3^Low^, score 1–3) (Fig. 7A and Figure S2). The expression of Foxa1 and Foxa2 was also scored using the same schema (Fig. 7B and C, and Figure S2). The clinicopathological features of HCC patients are shown in Supplementary Table 1. HDAC3 was expressed at low levels in 41.92% of male patients and 91.36% of female patients (*p* < 0.0001, chi-square test) (Fig. 7D). Therefore, HDAC3 expression was presumably lower in female HCC. It was noted that approximately 80% of patients with low HDAC3 expression in women also had minimal Foxa1/2 expression. Intriguingly, correlation analysis revealed that Foxa1 (r = 0.5265; *p* < 0.0001) and Foxa2 (r = 0.5550; *p* < 0.0001) were positively associated with HDAC3 expression in female HCC patients (Fig. 7E and F). Thus, low Foxa1/2 expression was correlated with low HDAC3 expression in female HCC patients.

## Discussion

Although sex differences in HCC have been identified in rodents and humans, with a much lower prevalence in females, the processes involved are less well understood. In the study of HDAC3 in HCC, we identified a significant sex difference in spontaneous HCC in *HDAC3*^*LCKO*^ mice, with female mice having earlier spontaneous HCC and a higher incidence than males. Most crucially, HDAC3 deletion suppressed Foxa1/2 expression and reversed the pro-carcinogenic effects of oestrogen. This could explain why *HDAC3*^*LCKO*^ female mice develop HCC earlier. A previous study revealed that oestrogen protects females against hepatocarcinogenesis by suppressing IL-6 [17]. However, this was not the case in HDAC3 ablated mice because we found that IL-6 knockout did not remove sex differences in HCC but accelerate HCC development earlier. Although continued activation of the IL-6 signalling pathway exerts protumour effects, IL-6 is critical for regenerative hepatocyte proliferation and hepatoprotection under physiological conditions [29]. Mice lacking IL-6 loss grew normally [30] but had more hepatocyte damage and poor repair ability when challenged. Furthermore, *HDAC3*^*LCKO*^&*IL-6*^*−/−*^ female mice developed more and larger tumours than *HDAC3*^*LCKO*^ female mice, implying that HDAC3 deletion may amplify the negative effects of IL-6 deficiency. These findings suggest that IL-6 is a predominantly protective agent for hepatocytes. Therefore, we concluded that IL-6 is not a key regulator of sex differences in HCC in *HDAC3*^*LCKO*^ mice. In women, oestrogen promotes the development of breast cancer while preventing the progression of liver cancer^9^. Experimental evidence strongly suggests that ERα plays a role in female resistance to HCC carcinogenesis and that AR promotes carcinogenesis in males [10–13]. The precise mechanisms of how oestrogen plays the opposite role in cancer remain to be fully elucidated. Foxa1 and Foxa2 expression was drastically reduced in the liver tissues of *HDAC3*^*LCKO*^mice in our study. Furthermore, immunoprecipitation revealed no ER binding to Foxa1/2 in *HDAC3*^*LCKO*^ female mice. Foxa1/2 can modify female HCC resistance by regulating oestrogen signalling [22]. Therefore, the lack of Foxa1/2 caused by HDAC3 ablation might have released cancer promoting effect of oestrogen. Although we confirmed that knocking down HDAC3 in the liver reduced Foxa1/2, our previous ChIP-Seq data did not support the idea that HDAC3 regulates Foxa1/2 at the transcriptional level [16]. Thus, how HDAC3 regulates Foxa1/2 remains to be further explored. As we have shown here, oestrogen is procardiogenic in *HDAC3*^*LCKO*^ mice, suggesting that antioestrogen therapy, such as ovariectomy, might benefit female HCC patients with low HDAC3 levels, which still needs further investigation. In summary, our study, for the first time, reports that HDAC3 deficiency leads to a higher incidence and earlier onset of liver cancer in female mice. Our findings indicate that loss of HDAC3 promotes hepatocarcinogenesis in females by downregulating Foxa1/2 (as summarized in Fig. 7G). Our observations provide a novel direction for sex differences in HCC, and these insights might have unique implications for the prevention and treatment of HCC.

## Conclusion

In our study, we revealed that loss of HDAC3 reduces Foxa1/2 and thus promotes HCC development in females in an oestrogen-dependent manner. Our observations provide a novel direction for sex differences in HCC, and these insights might have unique implications for the prevention and treatment of HCC.

### Electronic supplementary material

Below is the link to the electronic supplementary material.

Supplementary Material 1

## Data Availability

The datasets used and/or analysed during the current study are available from the corresponding author on reasonable request.

## Abbreviations

ATCC: American Type Culture Collection
AR: androgen receptor
ASR: age-standardized rate
Bcl-2: B-cell lymphoma-2
CK19: Cytokeratin 19
DDR: DNA damage response
DMEM: Dulbecco’s modified Eagle’s medium
EMMA: European Mouse Mutant Archive
GS: Glutamine synthetase
HCC: Hepatocellular carcinoma
HBV: hepatitis B virus
HCV: hepatitis C virus
HDAC3: histone deacetylase 3
HPCs: hepatic progenitor cells
IL-6: interleukin-6
H3K9: K9 in histone H3
MMP2: matrix metallopeptidase 2
MMP9: matrix metallopeptidase 9
OCT: optimal cutting temperature
OVX: ovariectomy
PCNA: proliferating cell nuclear antigen
PLC: Primary liver cancer
PTPRO: protein tyrosine phosphatase receptor type O
ERα: receptor alpha
STR: short tandem repeat
