# Supplementary material for: Hepatocyte-specific HDAC3 ablation promotes hepatocellular carcinoma in females by suppressing Foxa1/2
Source: BMC Cancer. 2023 Sep 26;23:906. doi: 10.1186/s12885-023-11393-1 (PMC10521566; doi:10.1186/s12885-023-11393-1)
Supplement: Supplementary file 1 — Supplementary Material 1 [file 12885_2023_11393_MOESM1_ESM.docx]

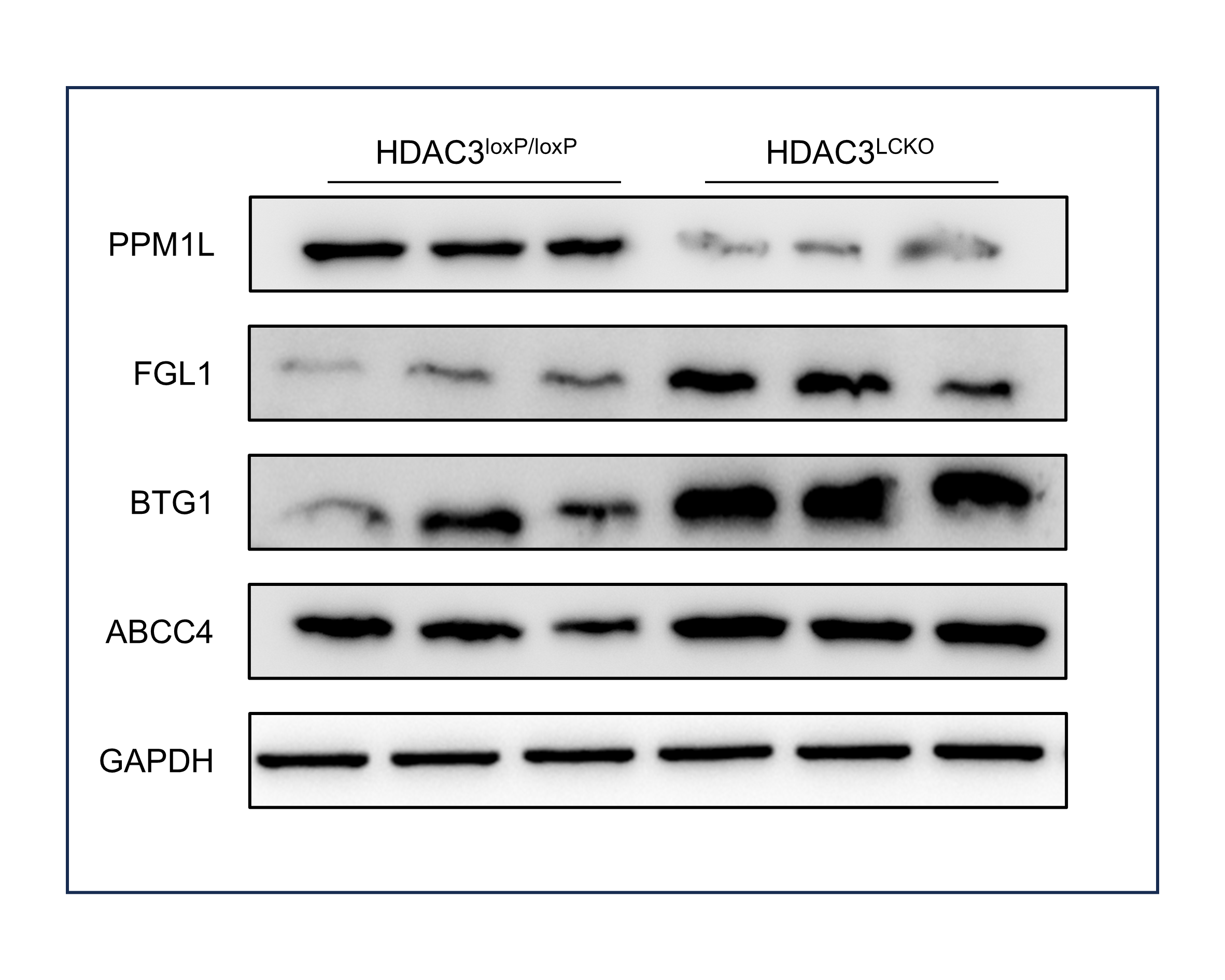


**Fig. S1.** Western Blot detection of PPM1L, FGL1, BTG1 and ABCC4 in *HDAC3^loxP/loxP^* and *HDAC3^LCKO^* livers.


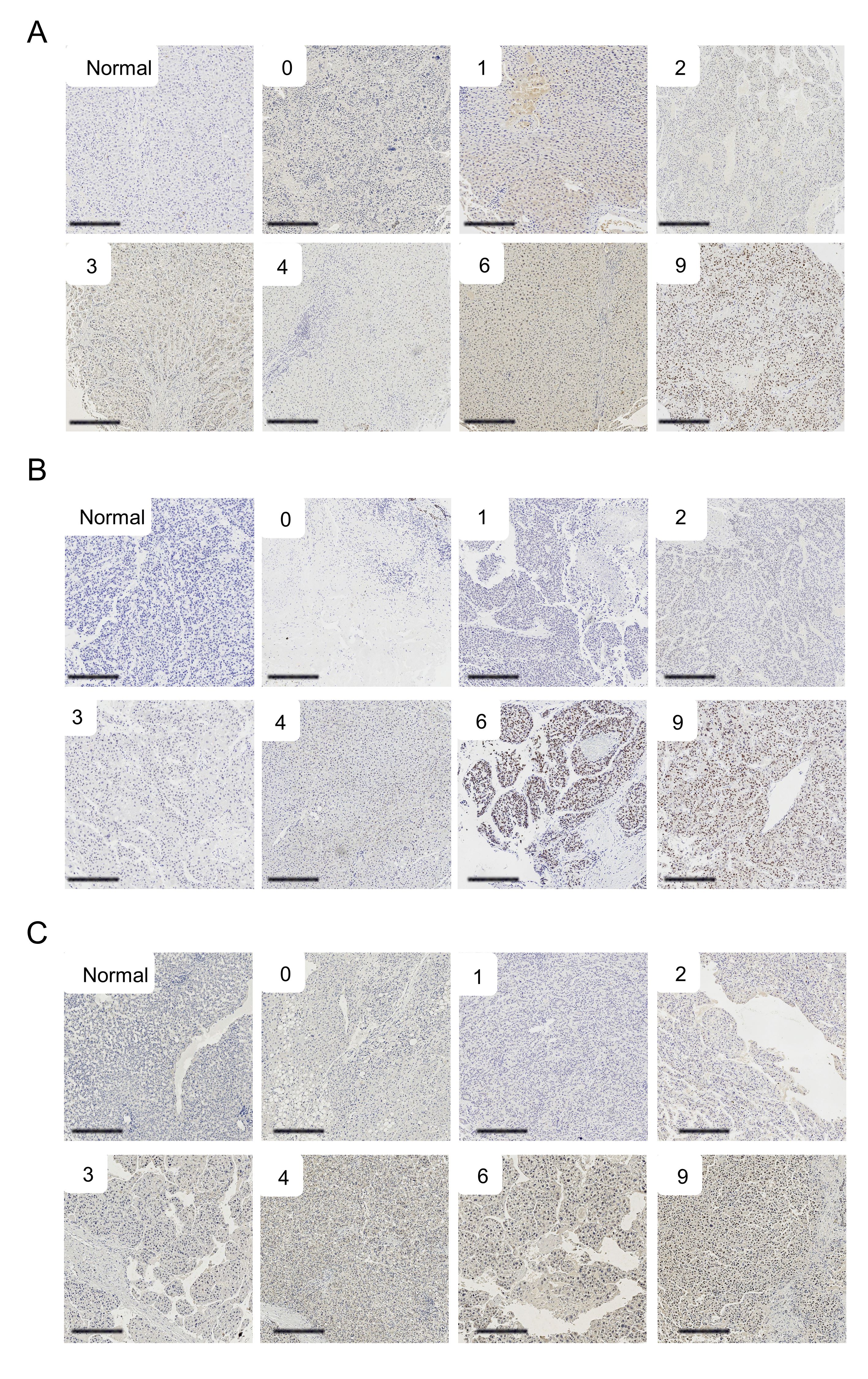


**Fig. S2.** IHC scores of HDAC3, Foxa1, and Foxa2 in 341 HCC patients. (A-C) According to the percentage of HDAC3^+^, Foxa1^+^, and Foxa2^+^ tumour cells in specific areas, specimens were assigned to the following categories: 0, <5%; 1, 5%-25%; 2, 25%-70%; 3, >75%. The intensity of IHC staining was categorized as follows: 1, weak; 2, moderate; and 3, intense. The final score of each specimen was the product of the percentage of HDAC3^+^, Foxa1^+^, and Foxa2^+^ tumour cells multiplied by the intensity of IHC staining. Therefore, the potential final scores were 0, 1, 2, 3, 4, 6, and 9, and the corresponding figures are presented. An IHC score of 0, 1, 2, or 3 was considered a low expression. A score of 4, 6, or 9 was considered high expression. Scale bar, 100 μm.

**Table S1.** Correlations between HDAC3 expression and clinicopathological features of HCC patients.

| Total number | | | | HDAC3 expression | |  | *p* |
| --- | --- | --- | --- | --- | --- | --- | --- |
|  |  |  |  | Low (n = 283) | High (n = 58) |  |  |
| Gender |  |  |  |  |  |  |  |
| Male |  | 260 |  | 209 | 51 |  | 0.0217* |
| Female |  | 81 |  | 74 | 7 |  |  |
| Year (yr) |  |  |  |  |  |  |  |
| ≤ 60 |  | 79 |  | 67 | 12 |  | 0.6235 |
| > 60 |  | 262 |  | 216 | 46 |  |  |
| Tumor size (cm) |  |  |  |  |  |  |  |
| ≤ 6 |  | 174 |  | 143 | 31 |  | 0.6855 |
| > 6 |  | 167 |  | 140 | 27 |  |  |
| Tumor nodules |  |  |  |  |  |  |  |
| Solitary |  | 282 |  | 230 | 52 |  | 0.1242 |
| Multiple (≥ 2) |  | 59 |  | 53 | 6 |  |  |
| AFP (ng/ml) |  |  |  |  |  |  |  |
| ≤ 20 |  | 175 |  | 160 | 15 |  | 0.0001*** |
| > 20 |  | 166 |  | 123 | 43 |  |  |
| Cirrhosis |  |  |  |  |  |  |  |
| Yes |  | 208 |  | 158 | 50 |  | 0.0179** |
| No |  | 133 |  | 115 | 18 |  |  |
| Differentiate |  |  |  |  |  |  |  |
| Yes |  | 149 |  | 119 | 30 |  | 0.1760 |
| No |  | 192 |  | 164 | 28 |  |  |
| Satellite |  |  |  |  |  |  |  |
| Yes |  | 53 |  | 39 | 14 |  | 0.0473** |
| No |  | 288 |  | 244 | 44 |  |  |

**Table S2.** Antibodies used were listed in the manuscript.

| Antibody | Source | Application | Catalog | Dilutions |
| --- | --- | --- | --- | --- |
| GS | Abcam | IHC | # ab64613 | 1:200 |
| CK19 | Abcam | IHC | # ab133496 | 1:500 |
| HDAC3 | Abcam | WB  IHC | # ab7030 | 1:2000  1:200 |
| HNF4α | Abcam | IHC | # ab41898 | 1:200 |
| ERα | CST | IP | # 8644 | 1:100 |
| Foxa1 | CST  HUABIO | WB/IP  IHC | # 53528  ET1702-89 | 1:1000/1:50  1:200 |
| Foxa2 | CST | WB/IP  IHC | # 8186 | 1:1000/1:50  1:200 |
| Histone H3 | CST | WB | # 4499 | 1:2000 |
| GAPDH | KangChen Biotech | WB | # KC-5G4 | 1:2000 |
| Ki67 | Thermo | IHC | # RM-9106-S1 | 1:200 |

**Western Blot**

Figure WB 1A


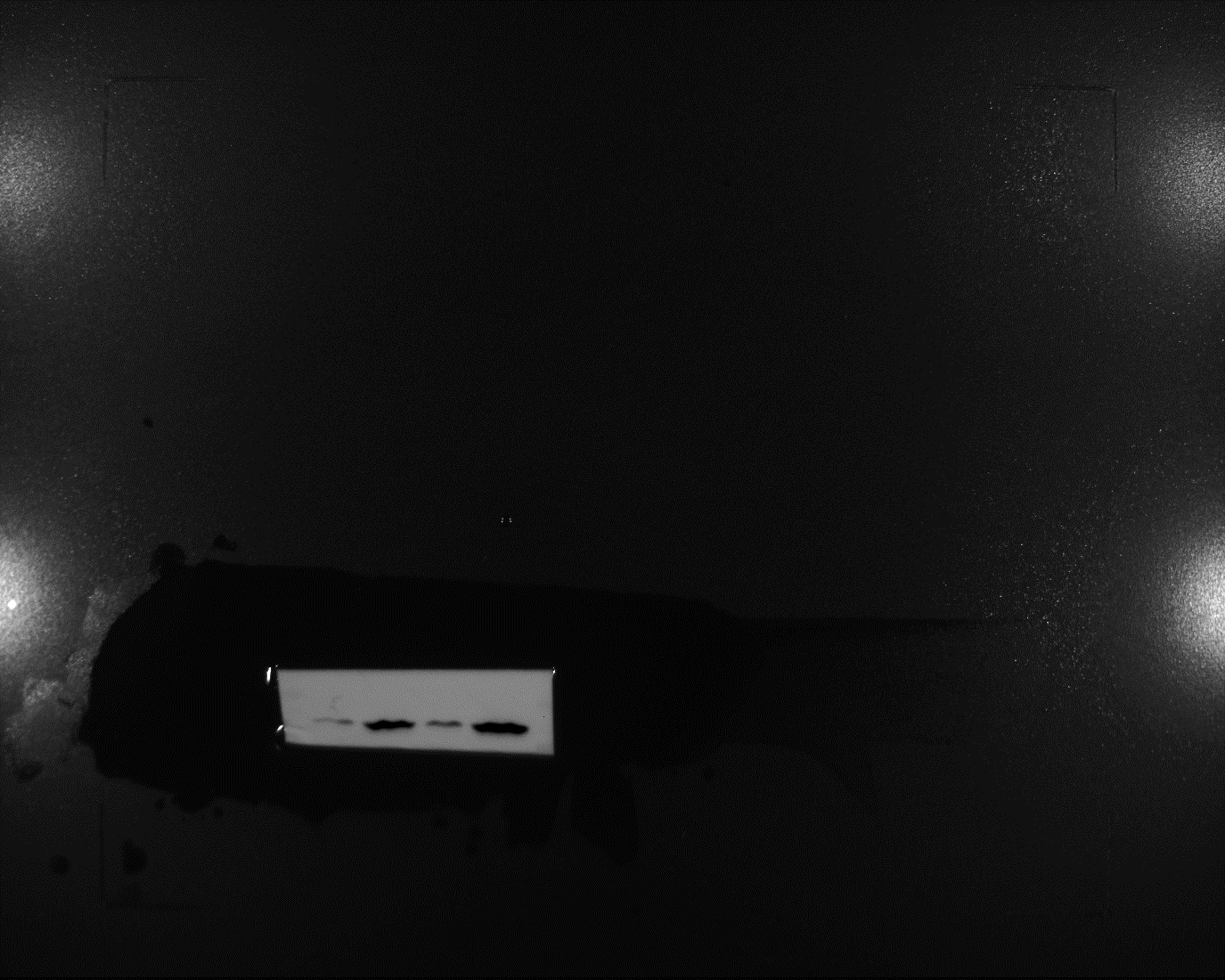


HDAC3


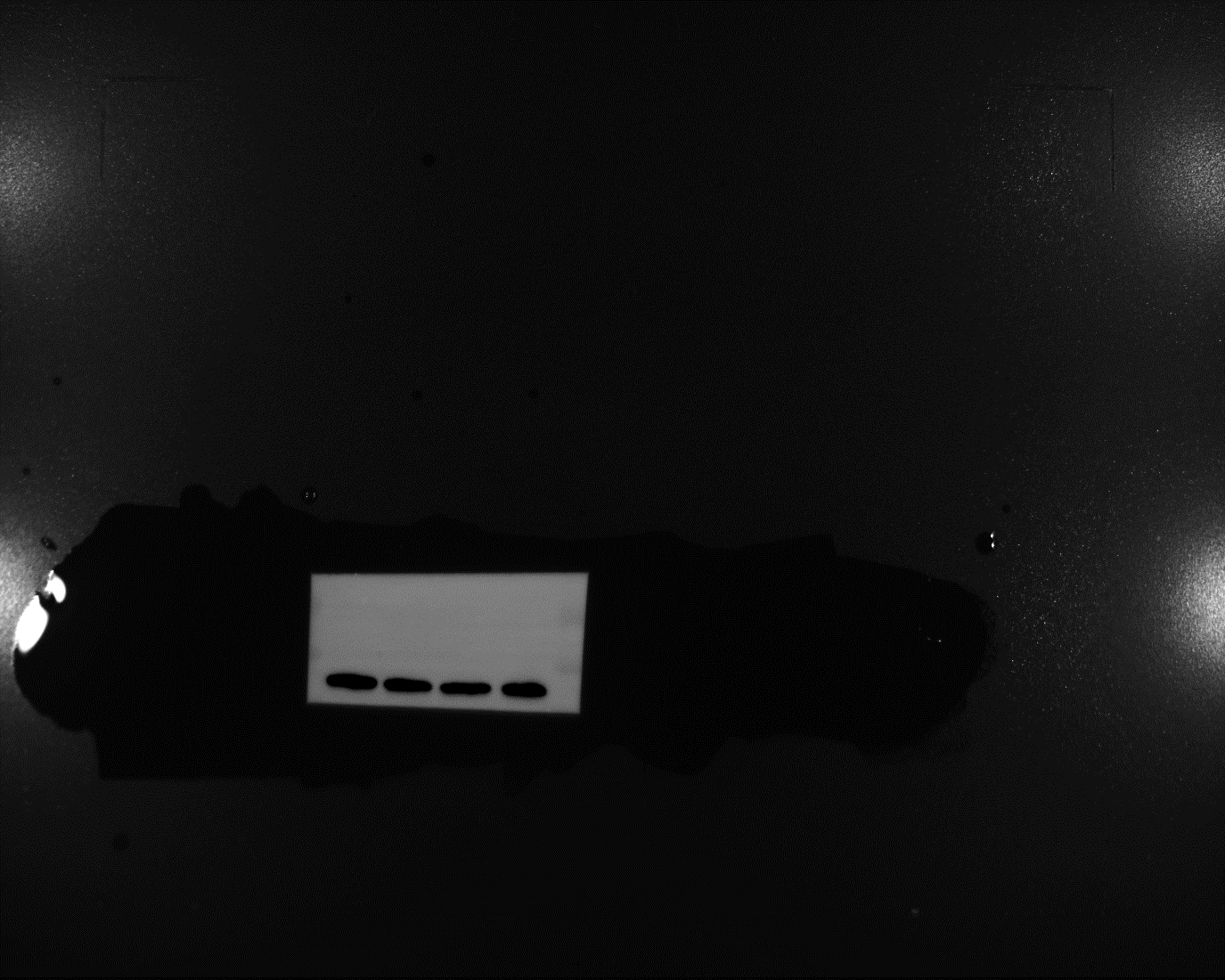


GAPDH


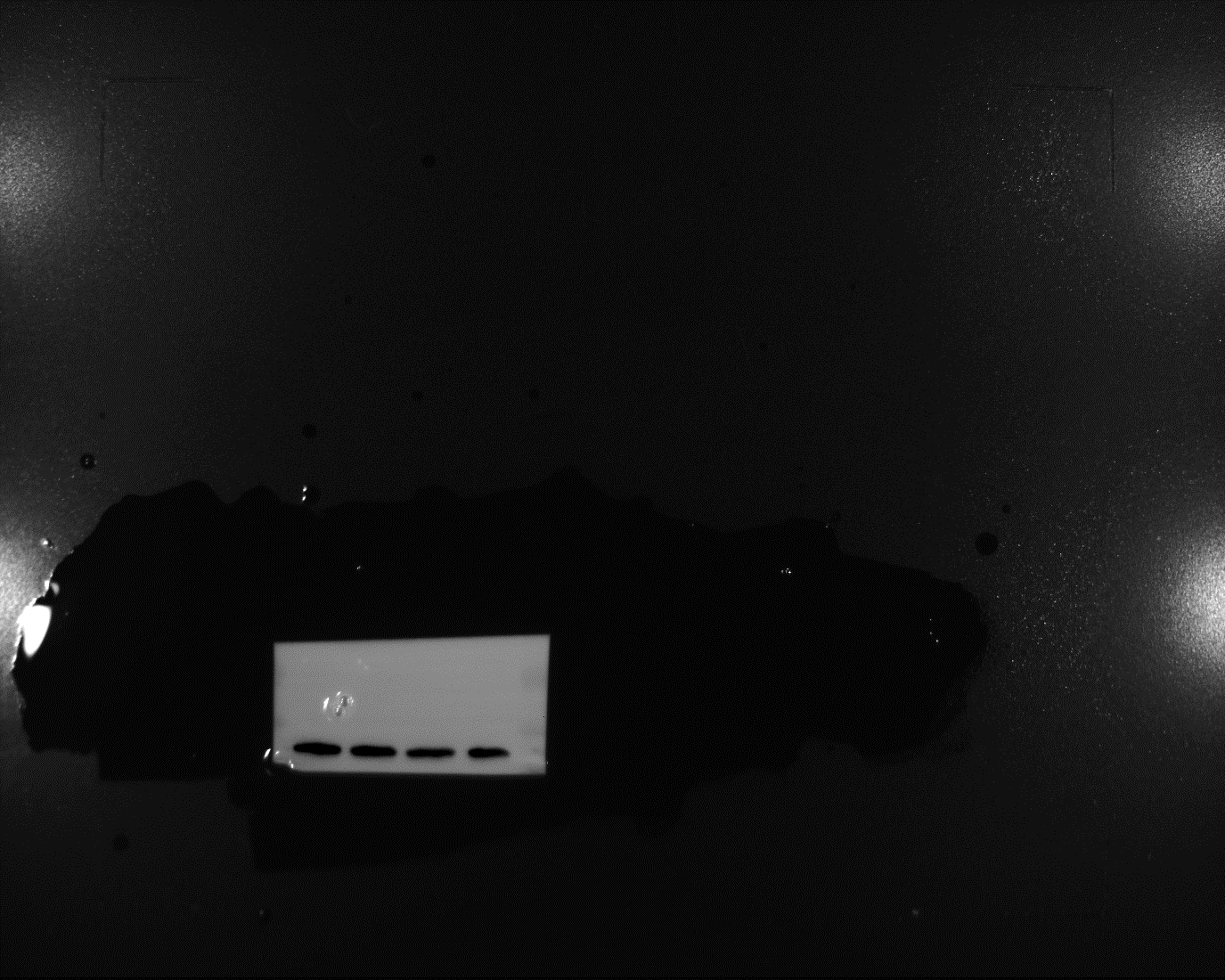


Histone H3

Figure WB 3B


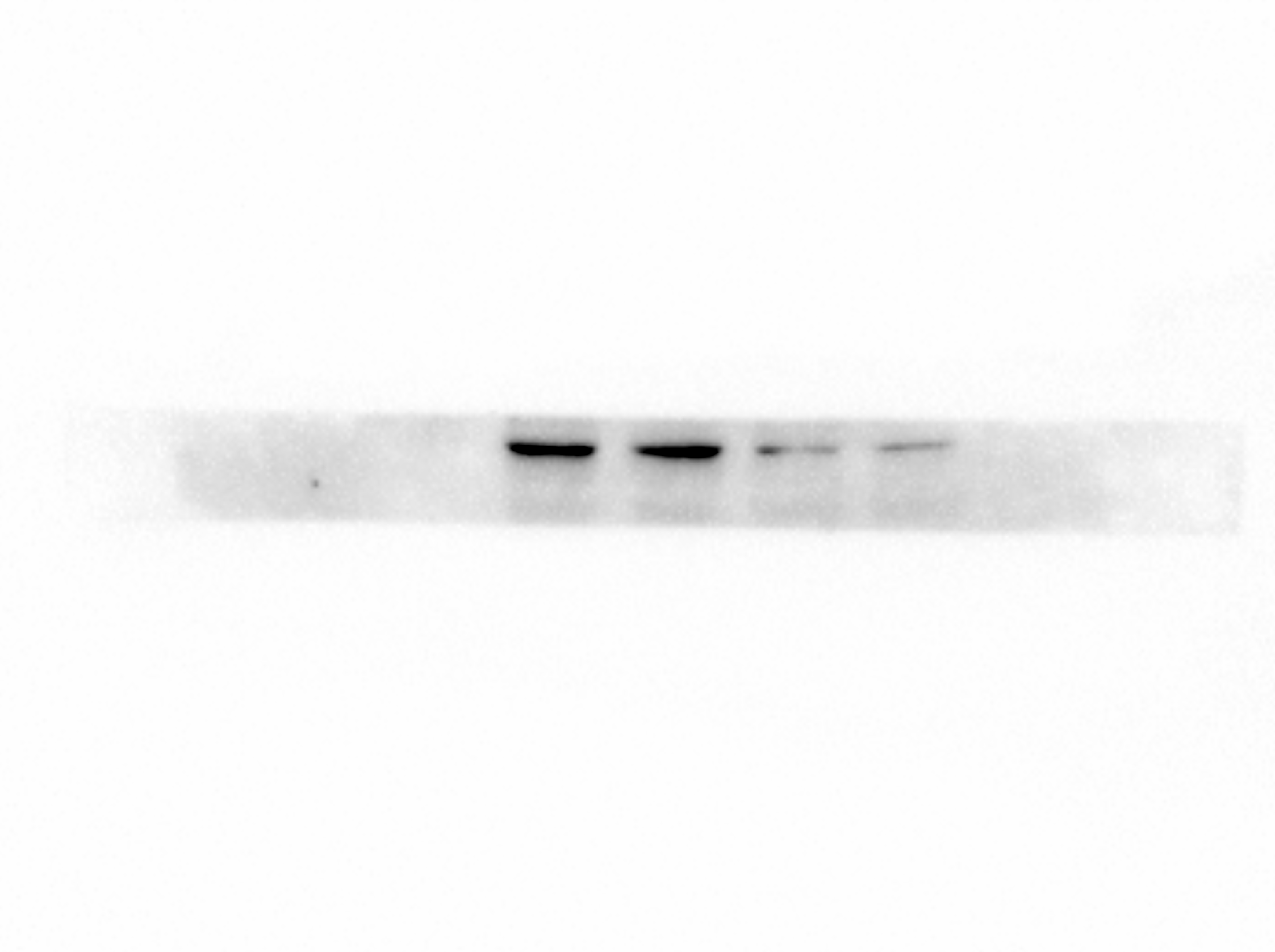


HDAC3


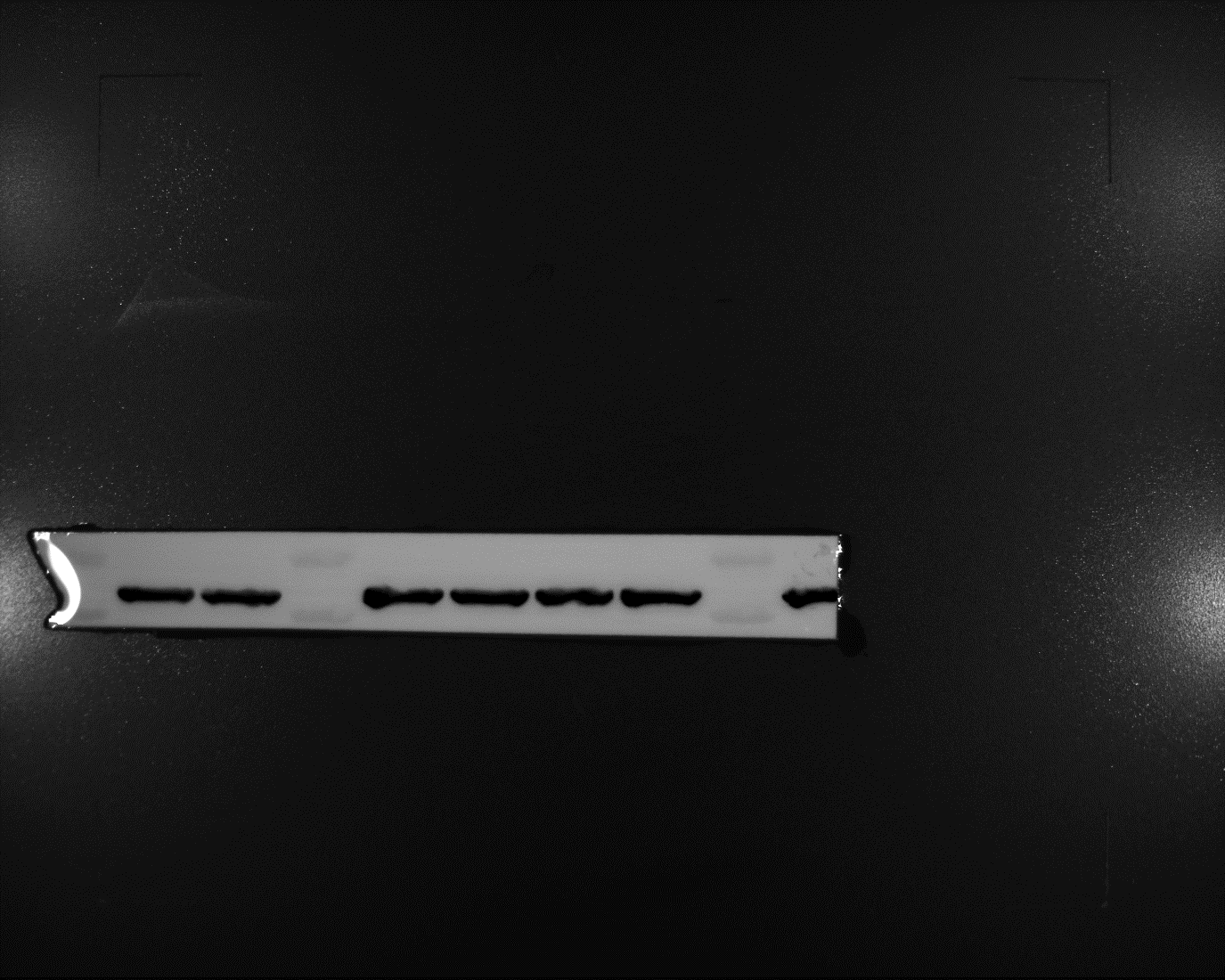


GAPDH


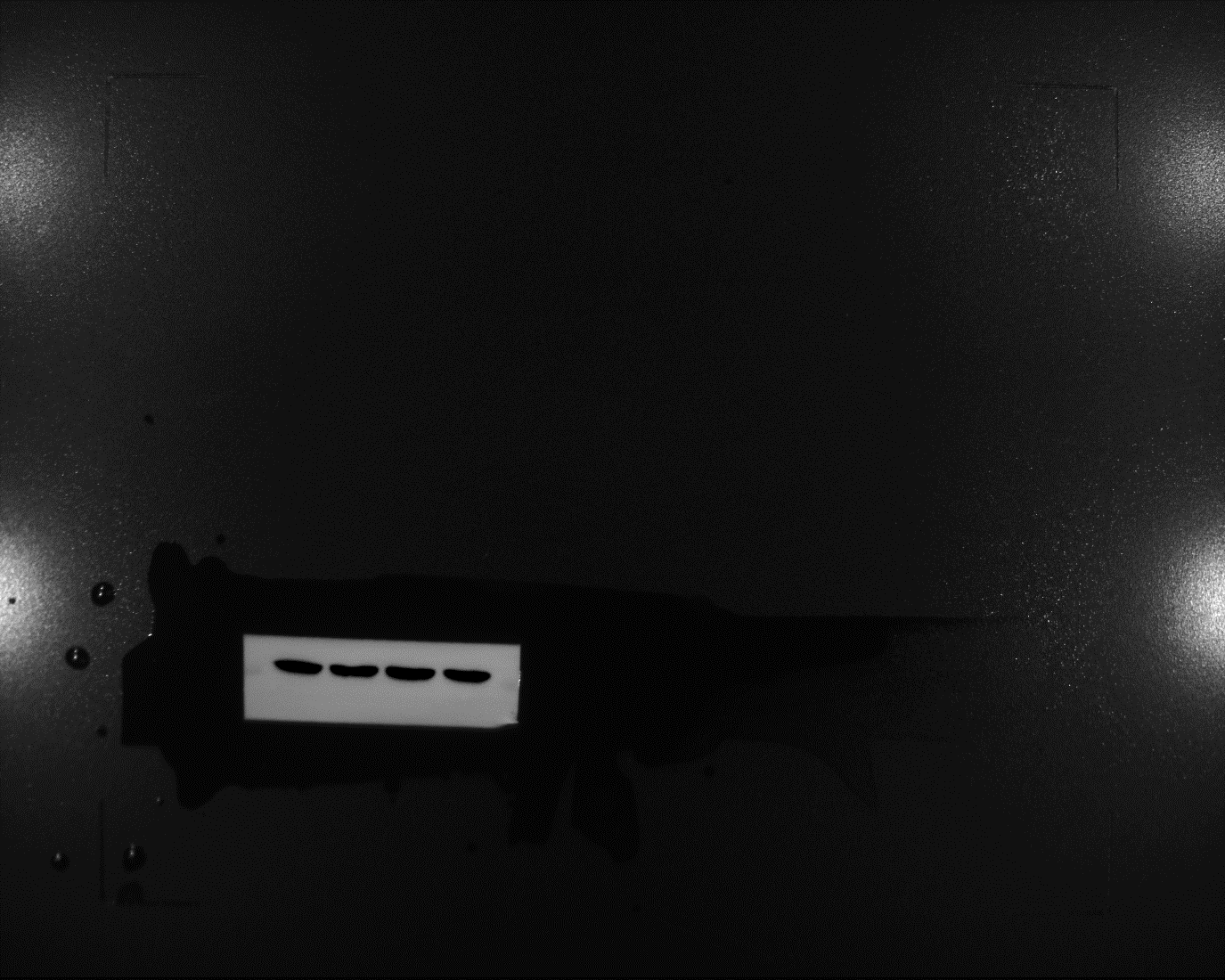


Histone H3

Figure WB 6B


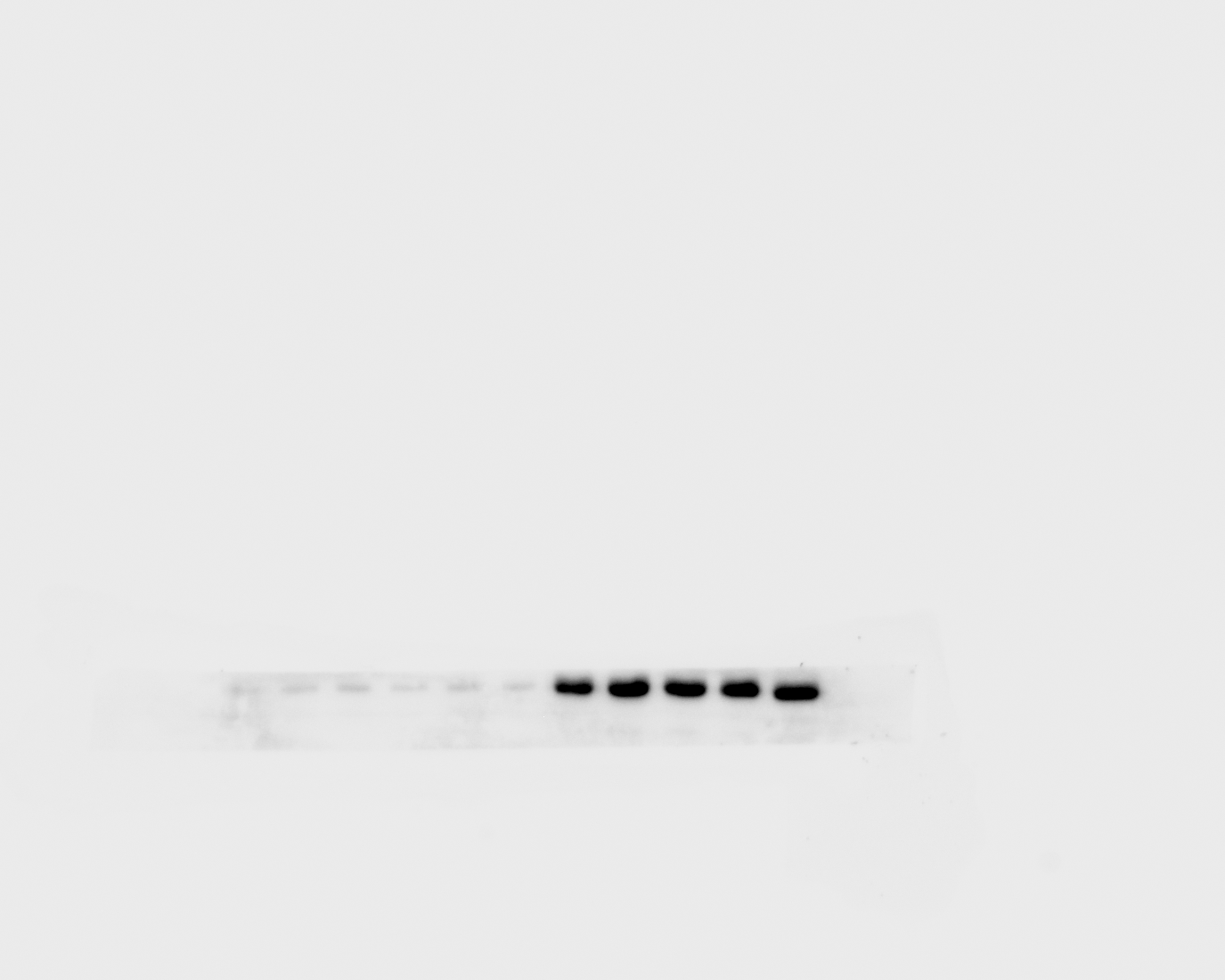


Male Foxa1


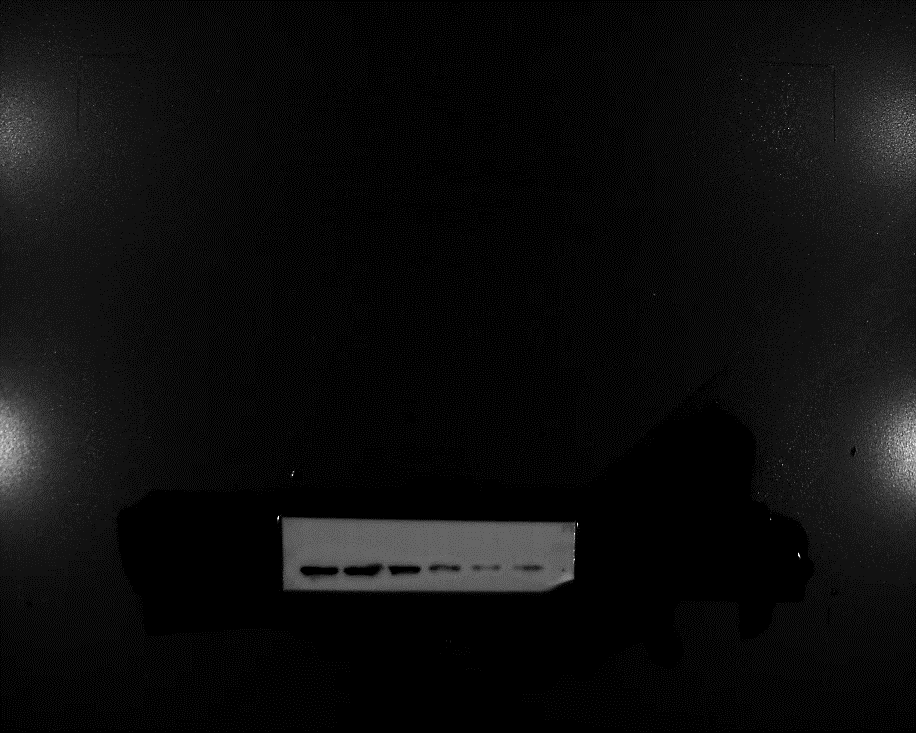


Male Foxa2


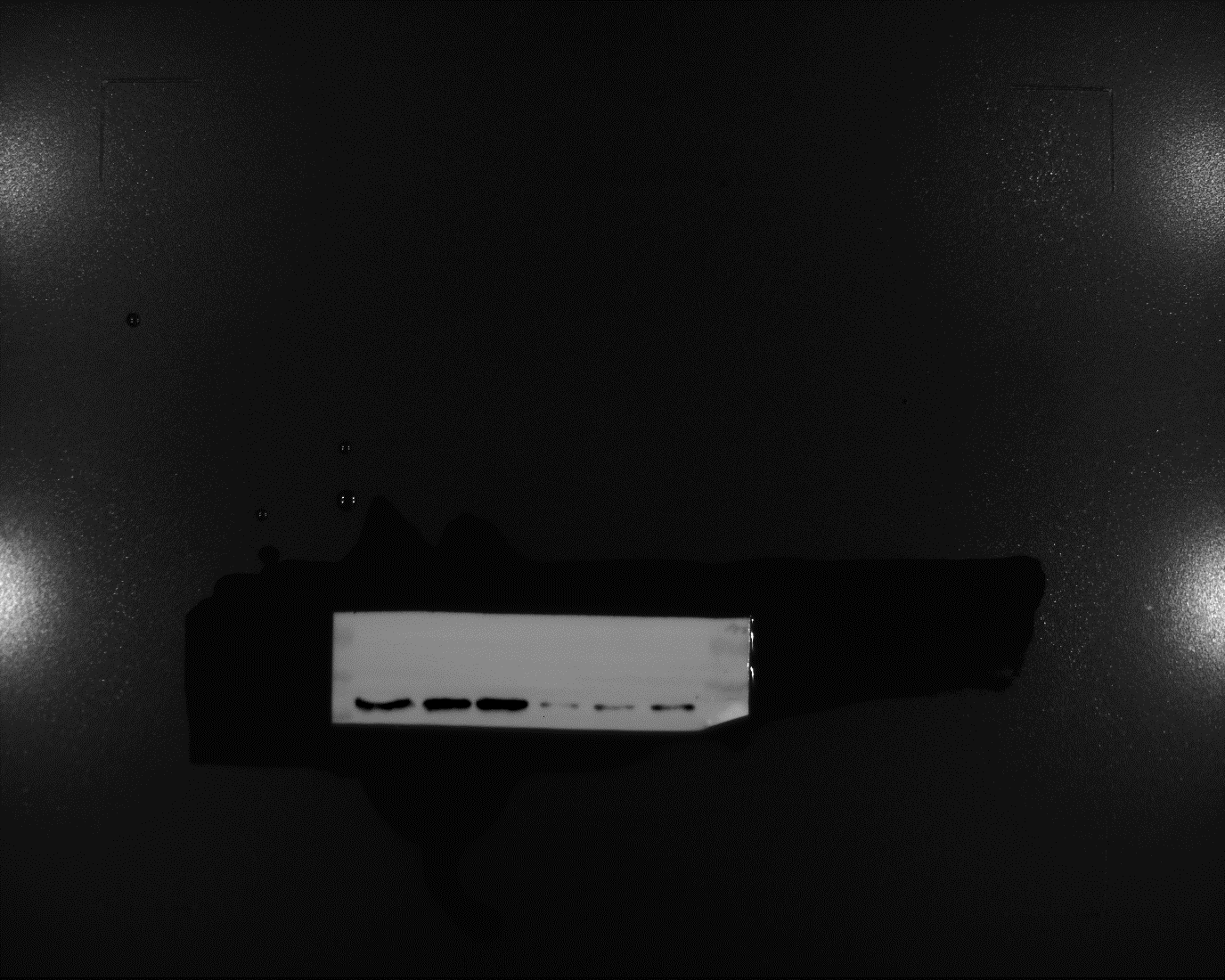


Male HDAC3


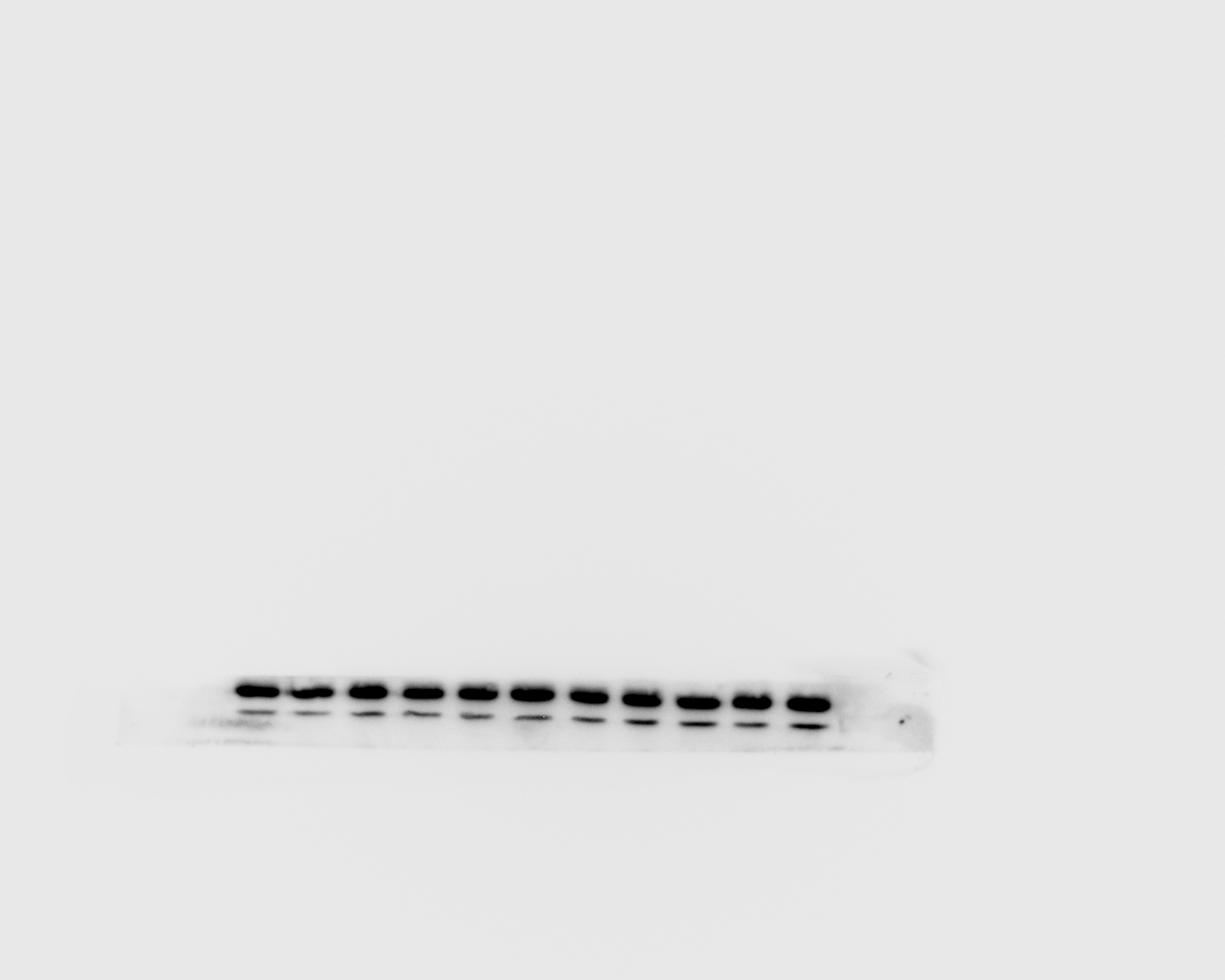


Male GAPDH


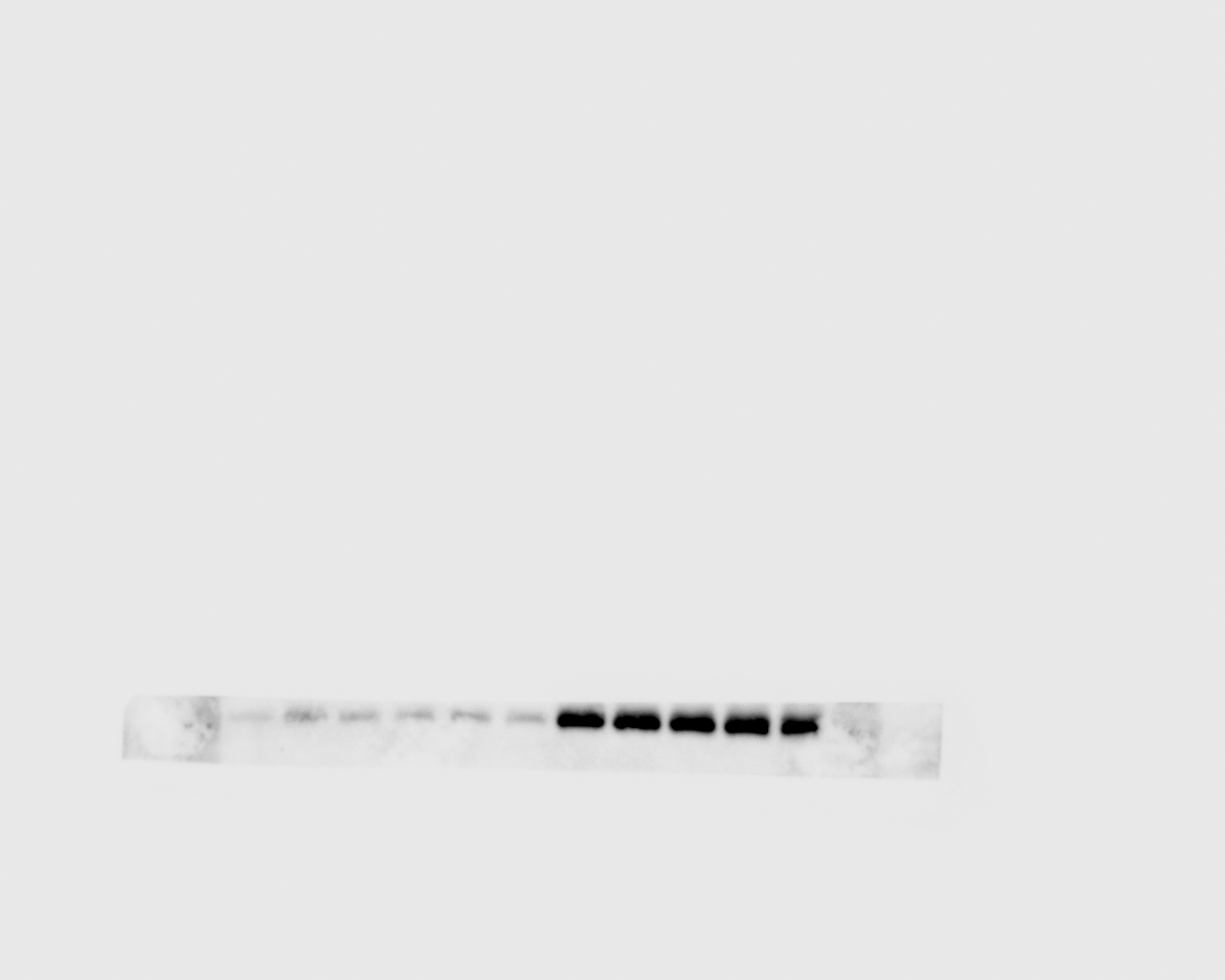


Female Foxa1


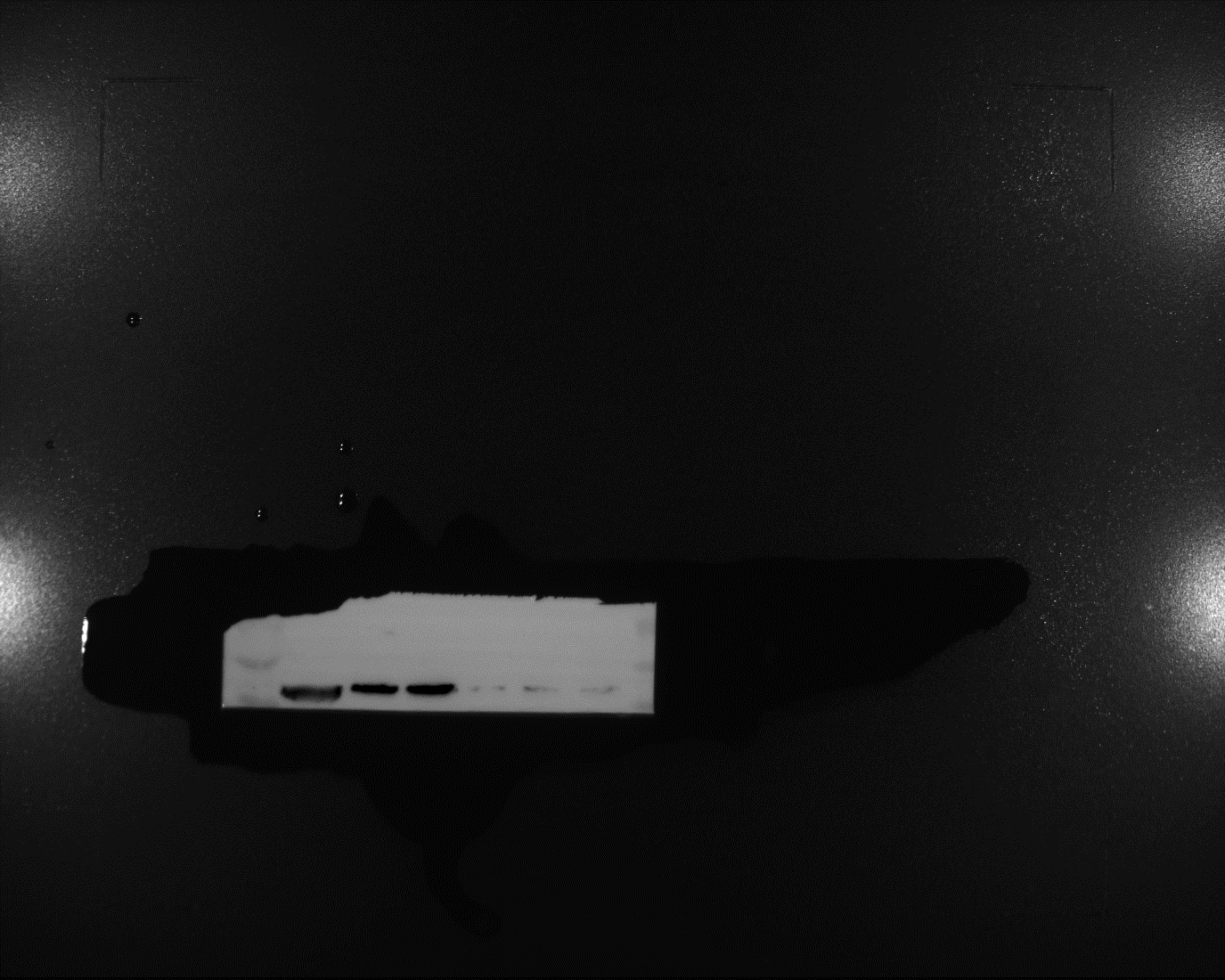


Female Foxa2


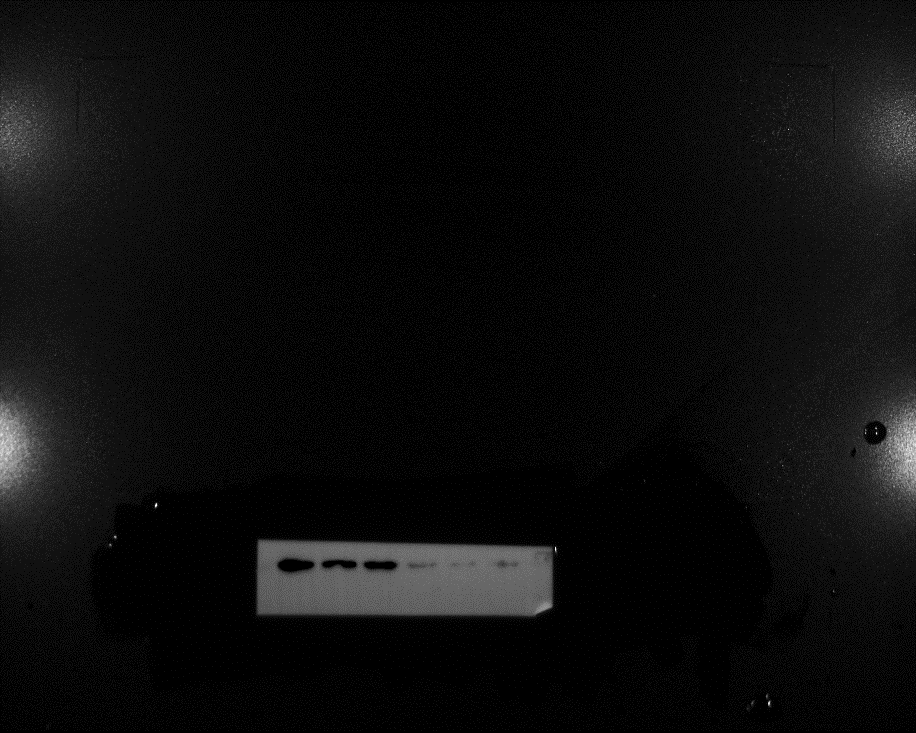


Female HDAC3


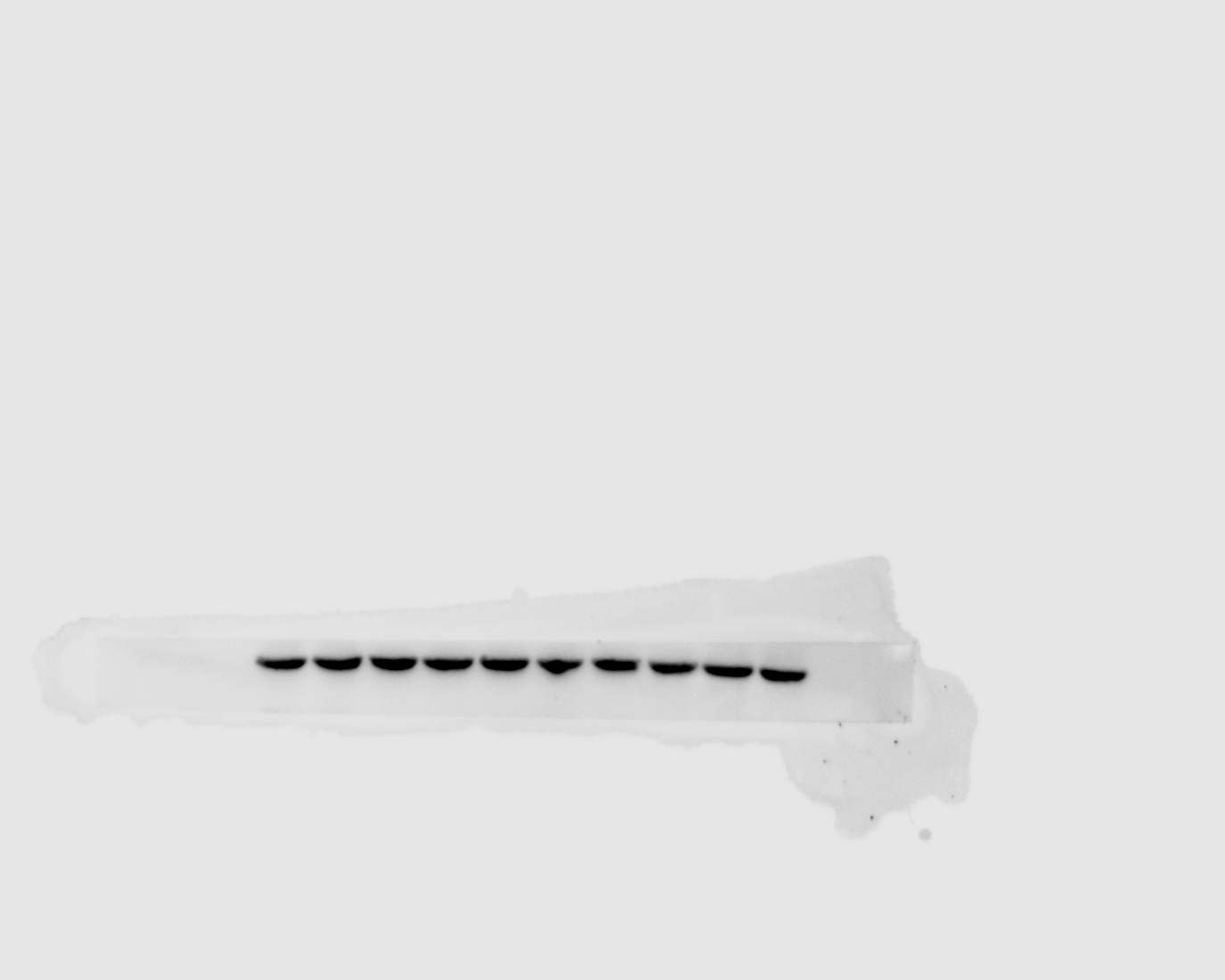


Female GAPDH

Figure WB 6C


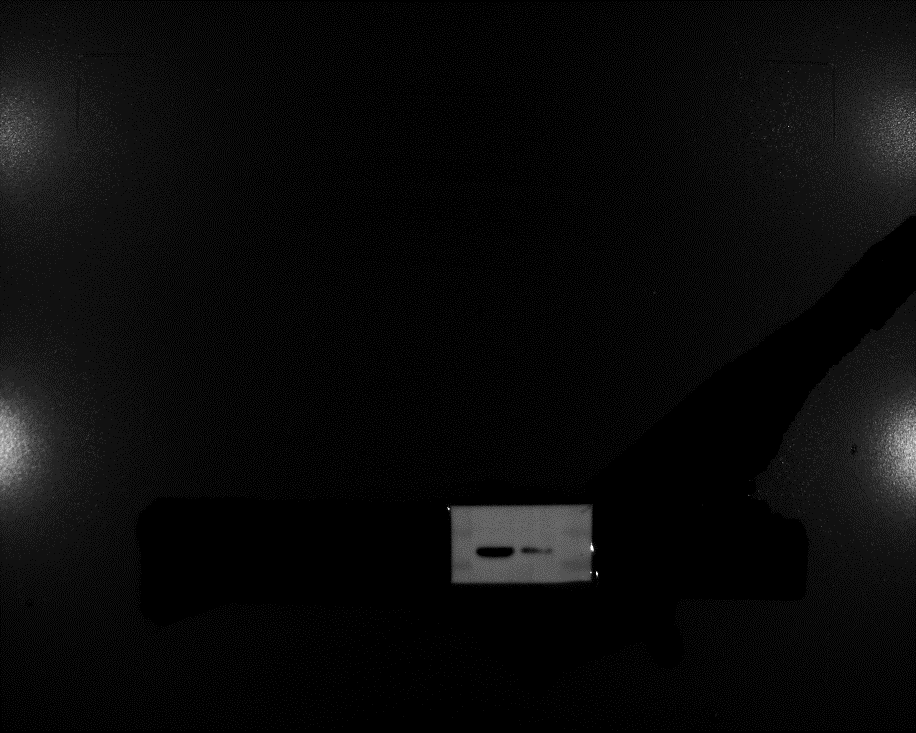


Foxa1


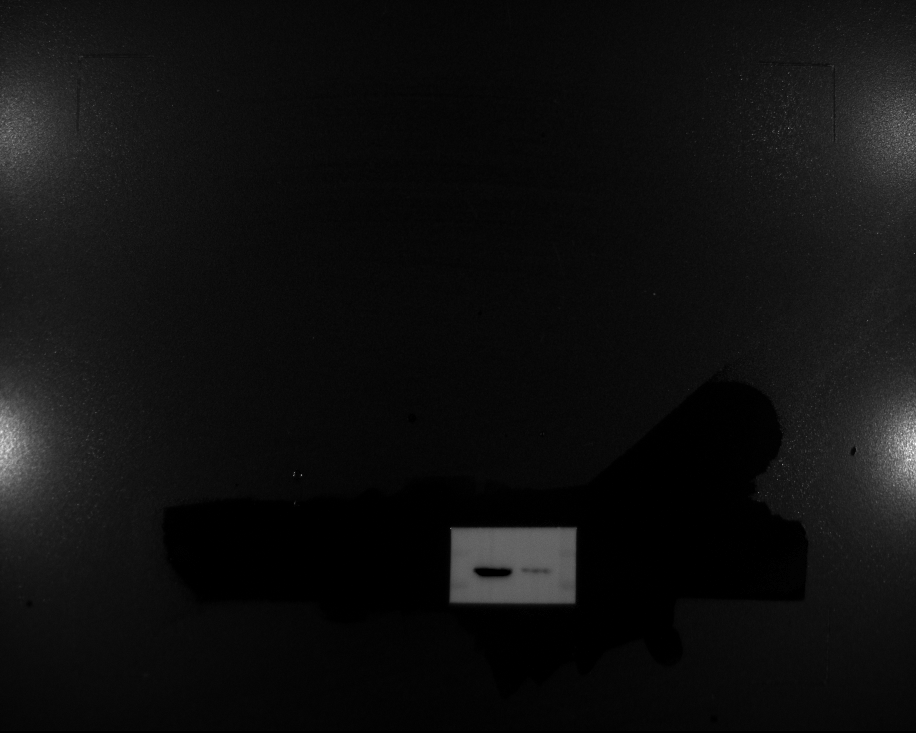


Foxa2


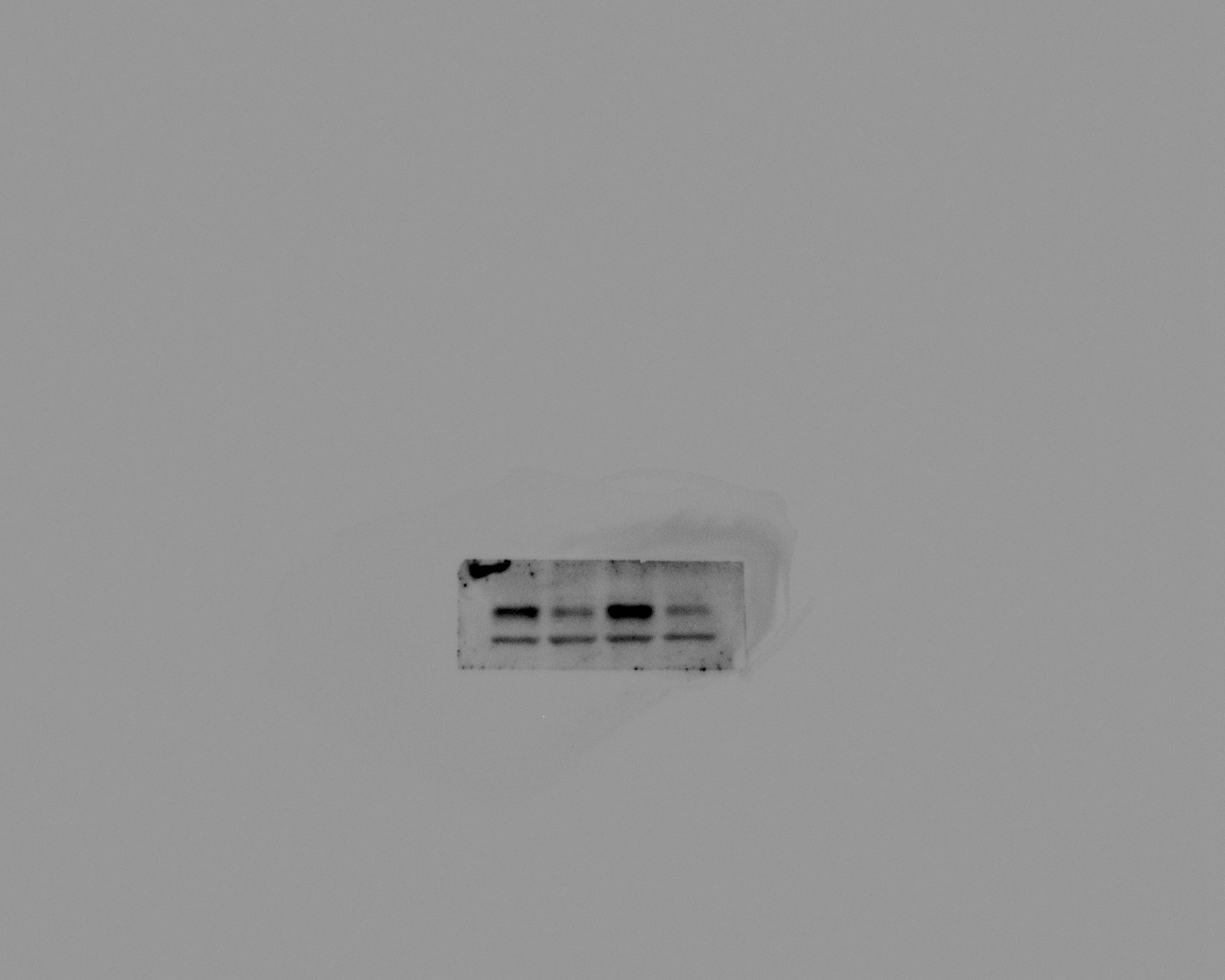


siHDAC3


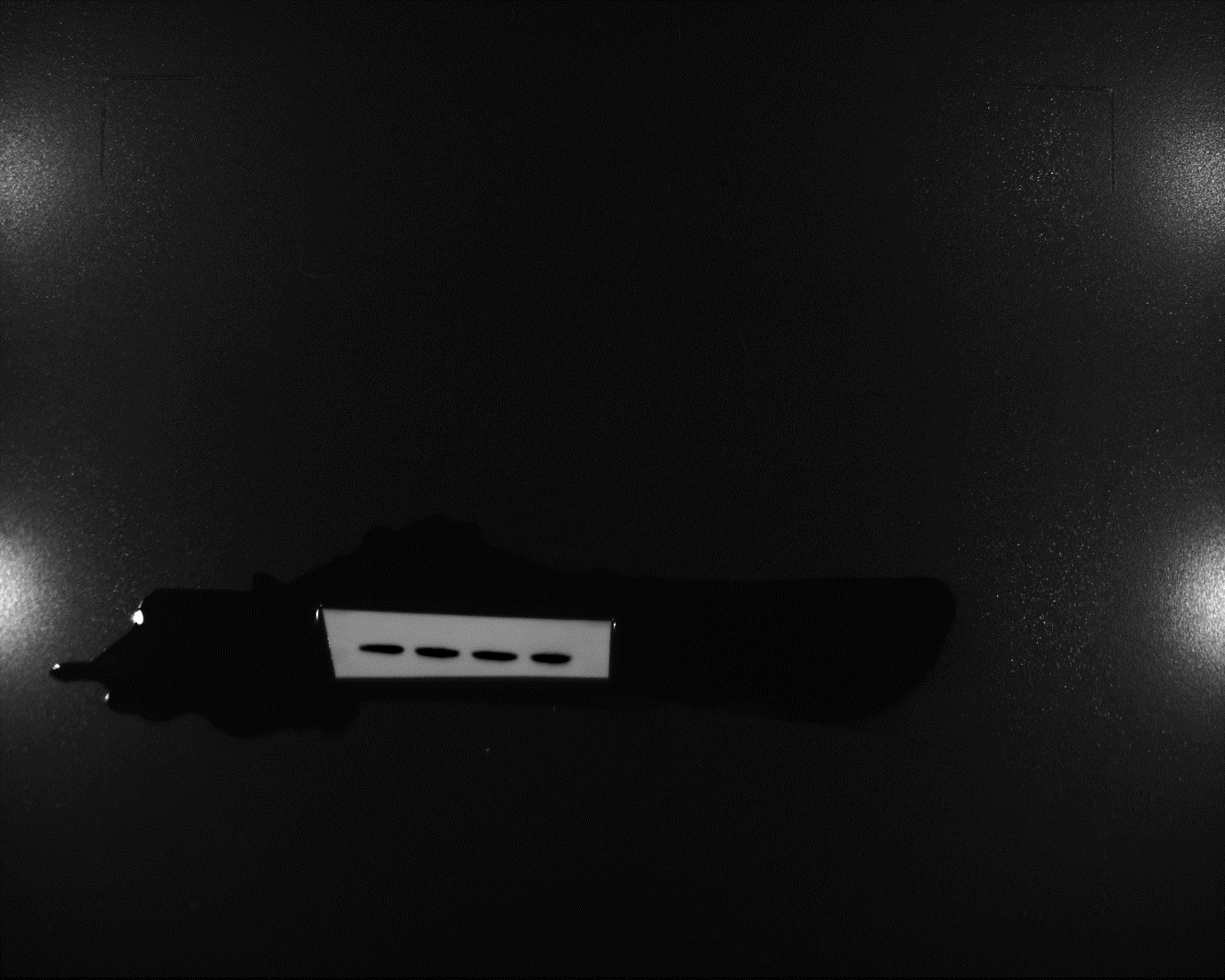


GAPDH

Figure WB 6D


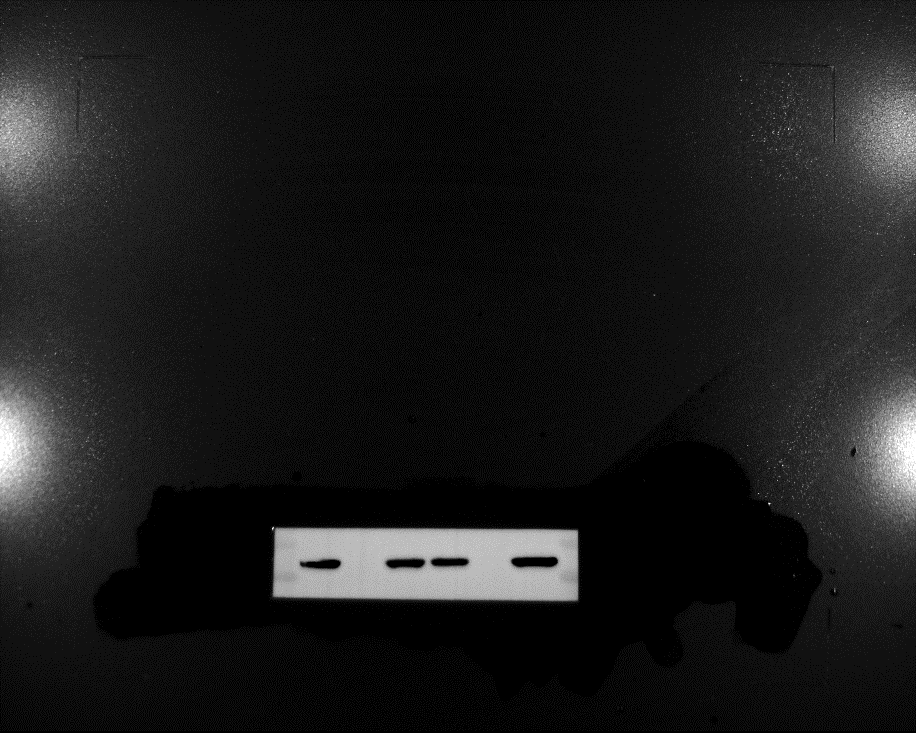


HDAC3^loxp^ Foxa1 ER


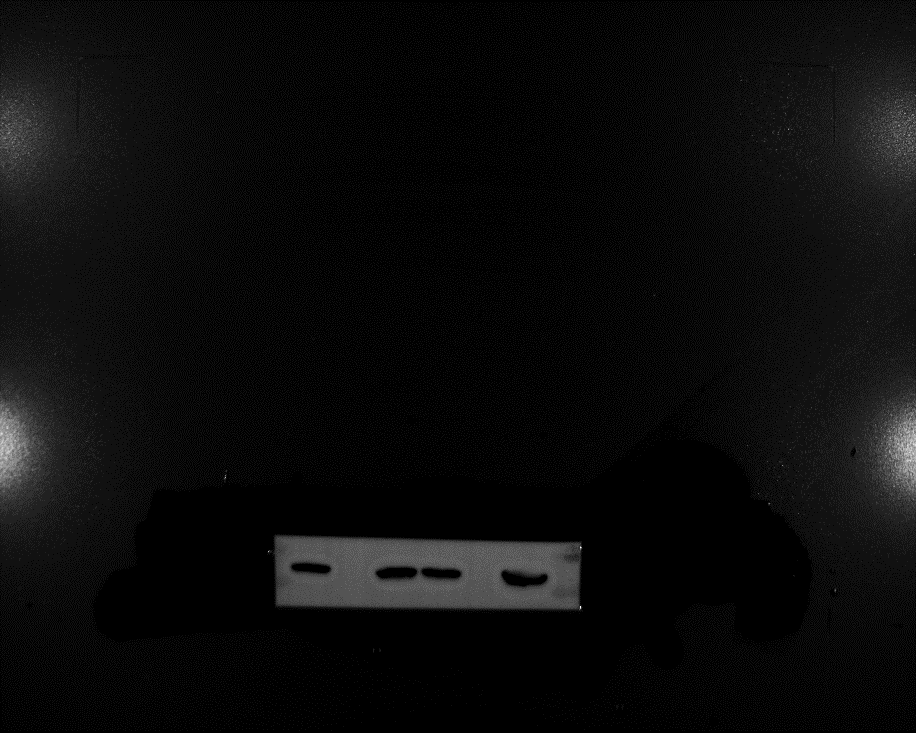


HDAC3^loxp^ ERα Foxa1


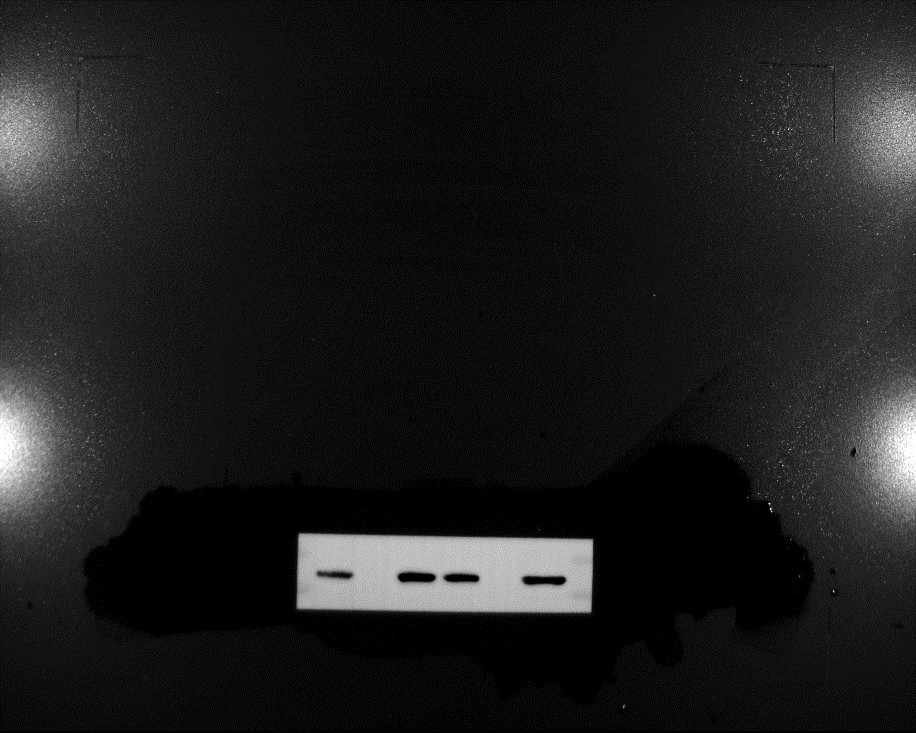


HDAC3^loxp^ Foxa2 ER


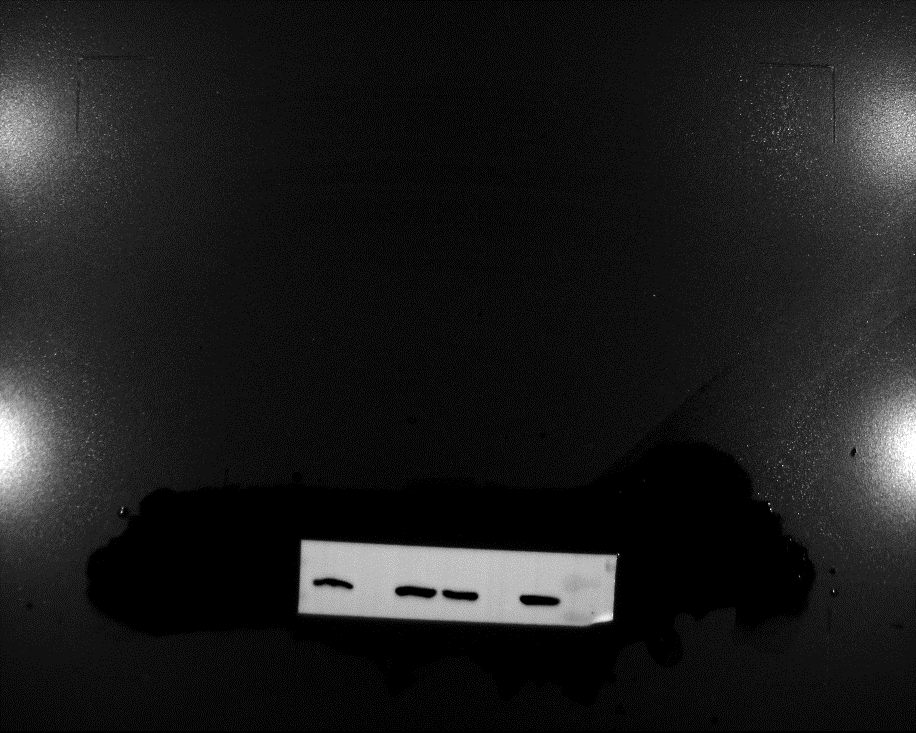


HDAC3^loxp^ ERα Foxa2


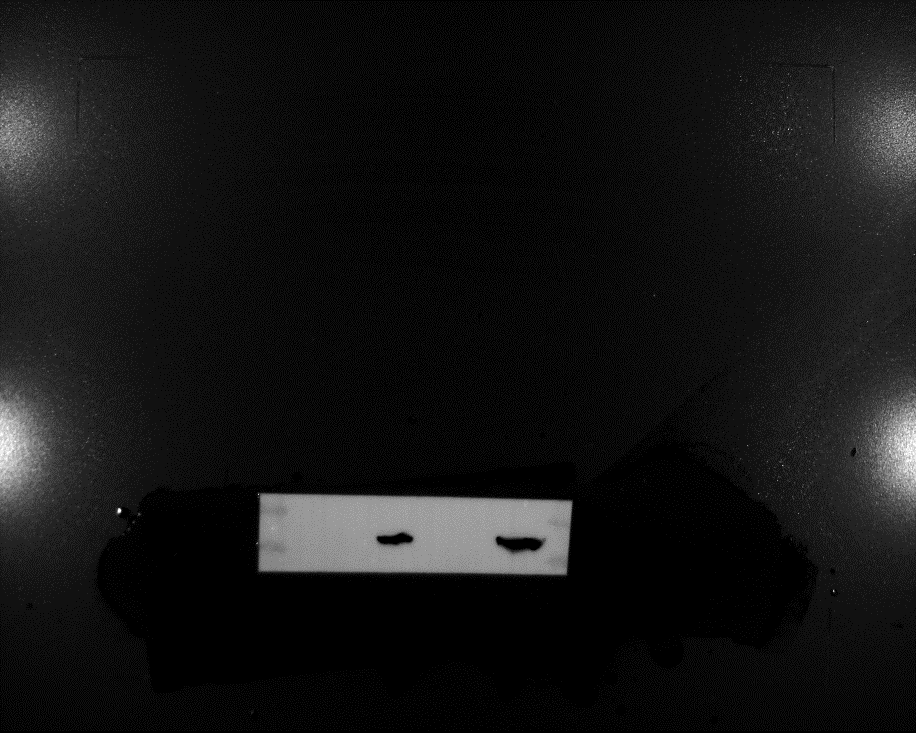


HDAC3^LCKO^ Foxa1 ER


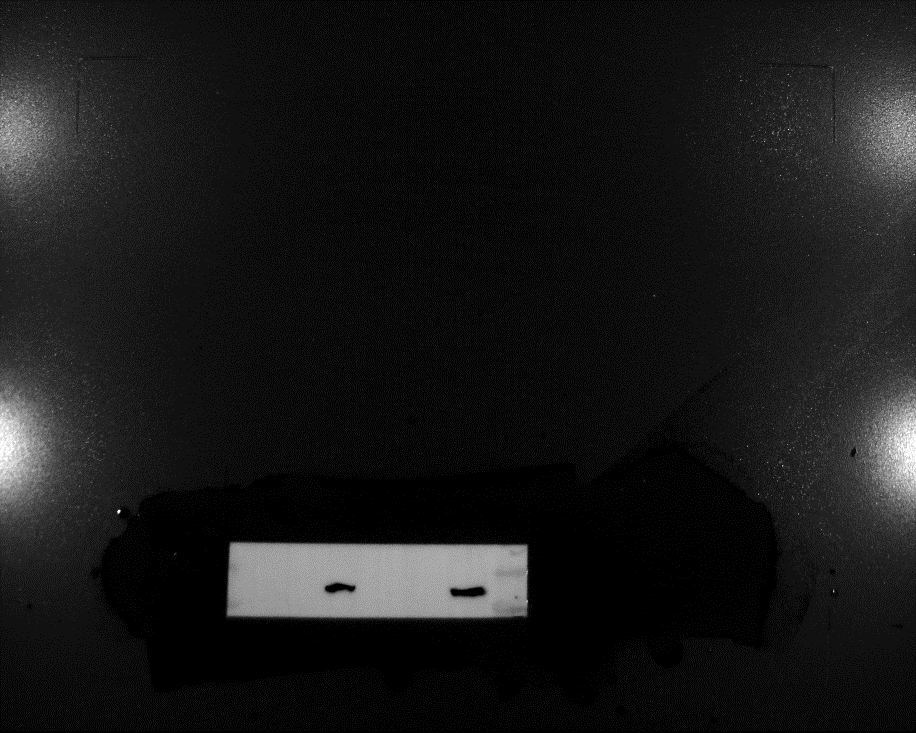


HDAC3^LCKO^ ER Foxa1


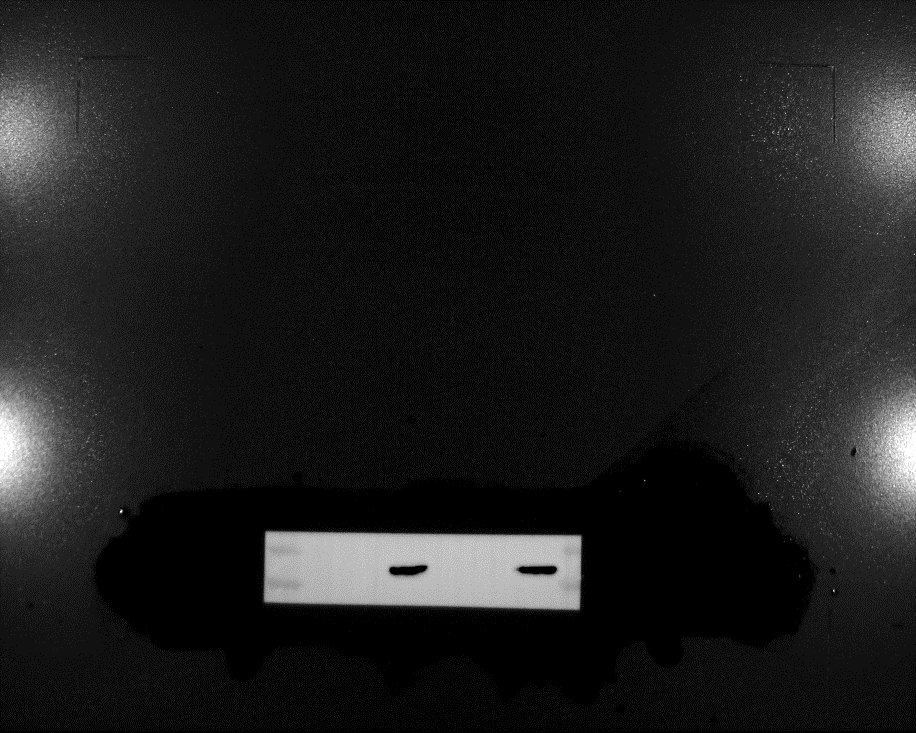


HDAC3^LCKO^ Foxa2 ER


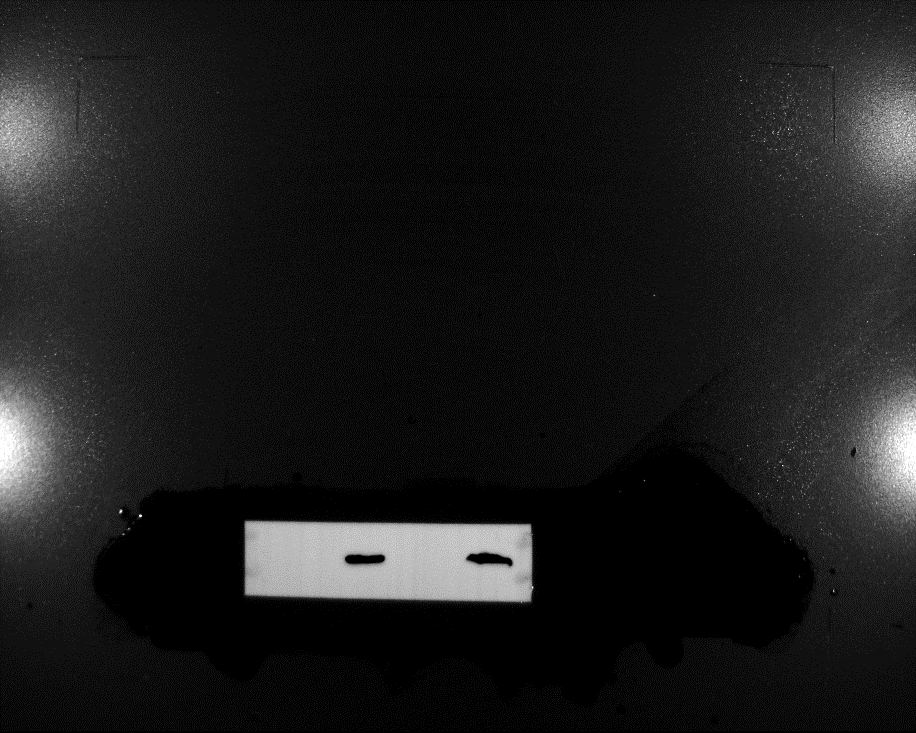


HDAC3^LCKO^ ER Foxa2

Figure WB 6E


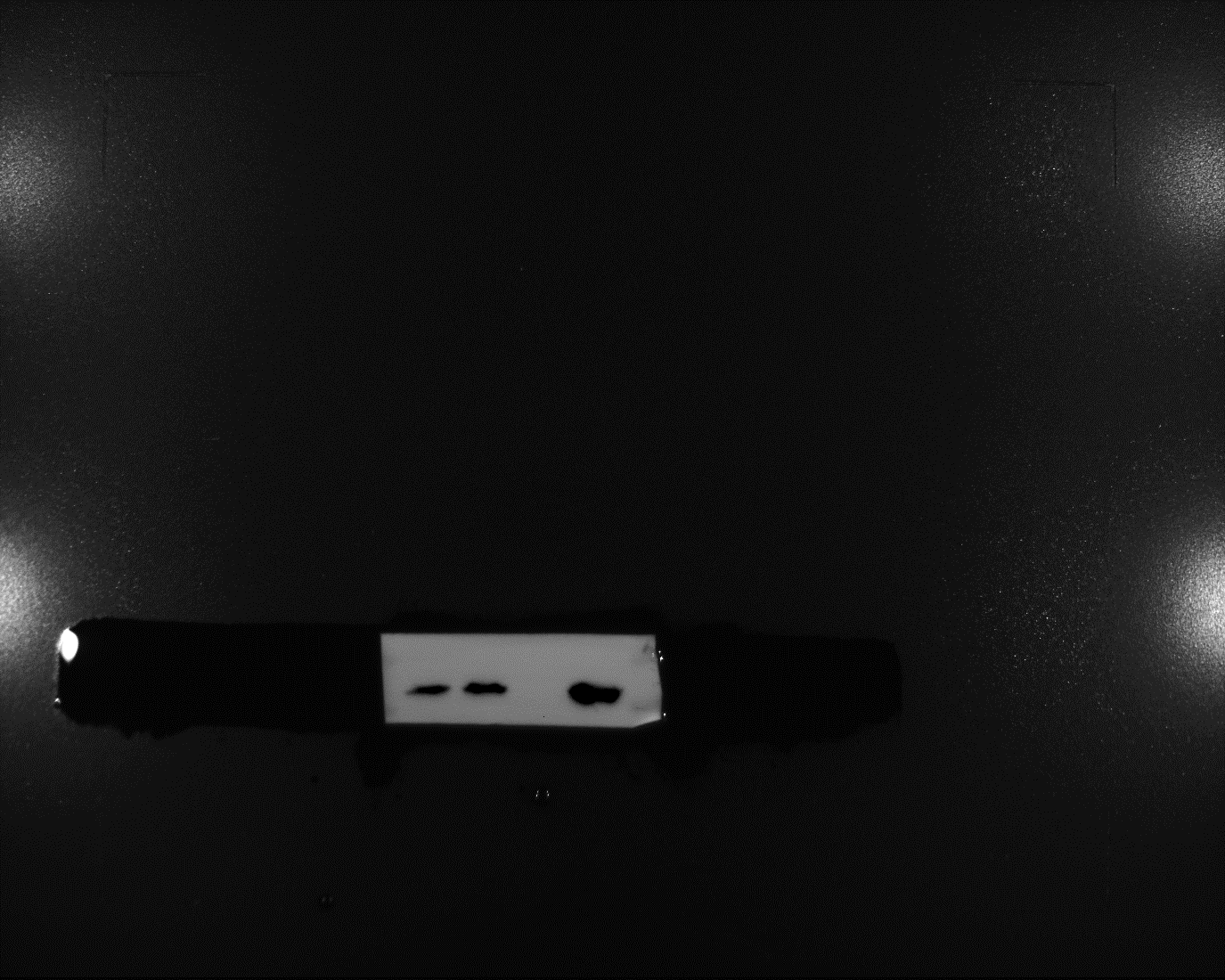


HDAC3^loxp^ AR Foxa1


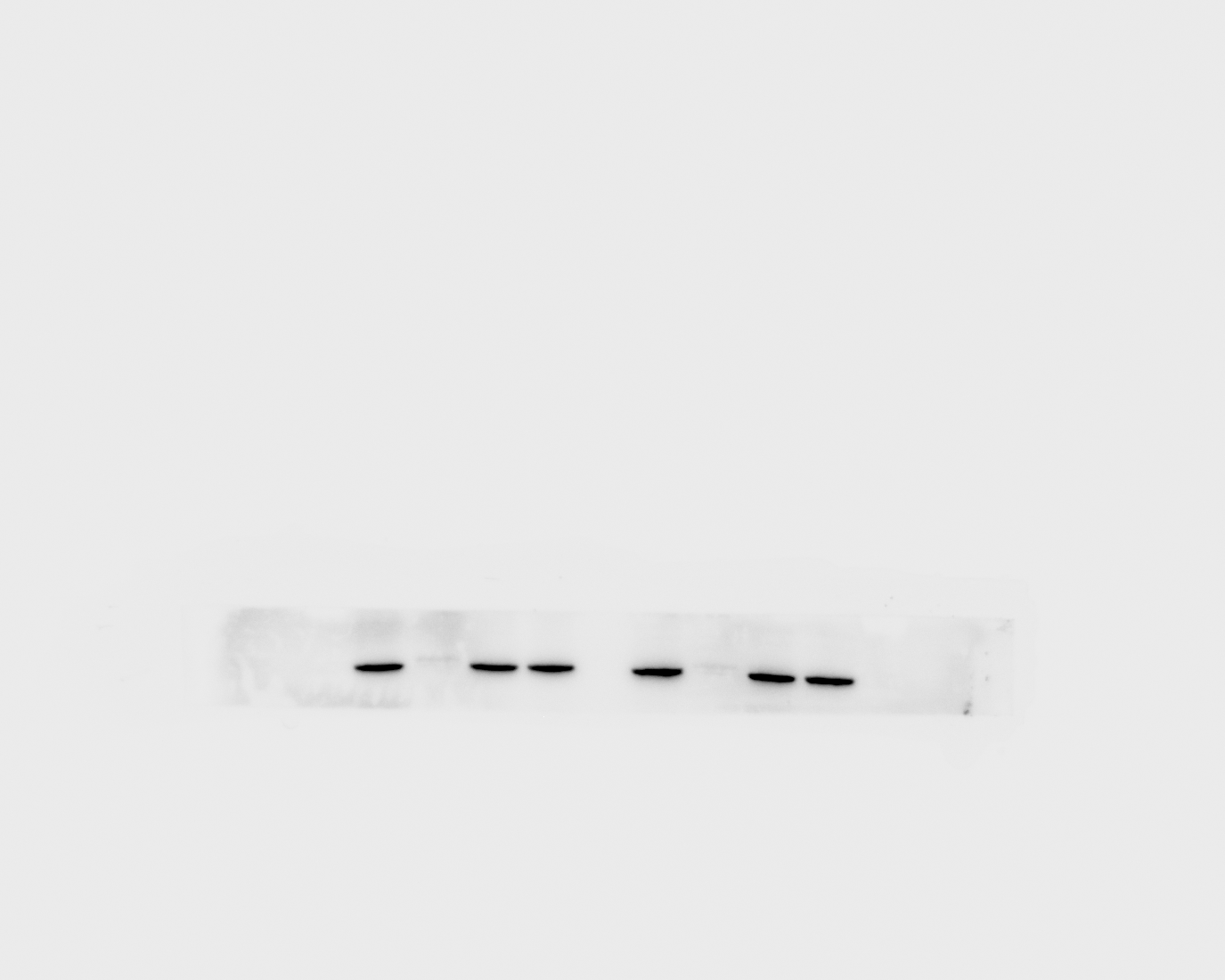


HDAC3^loxp^ AR Foxa2


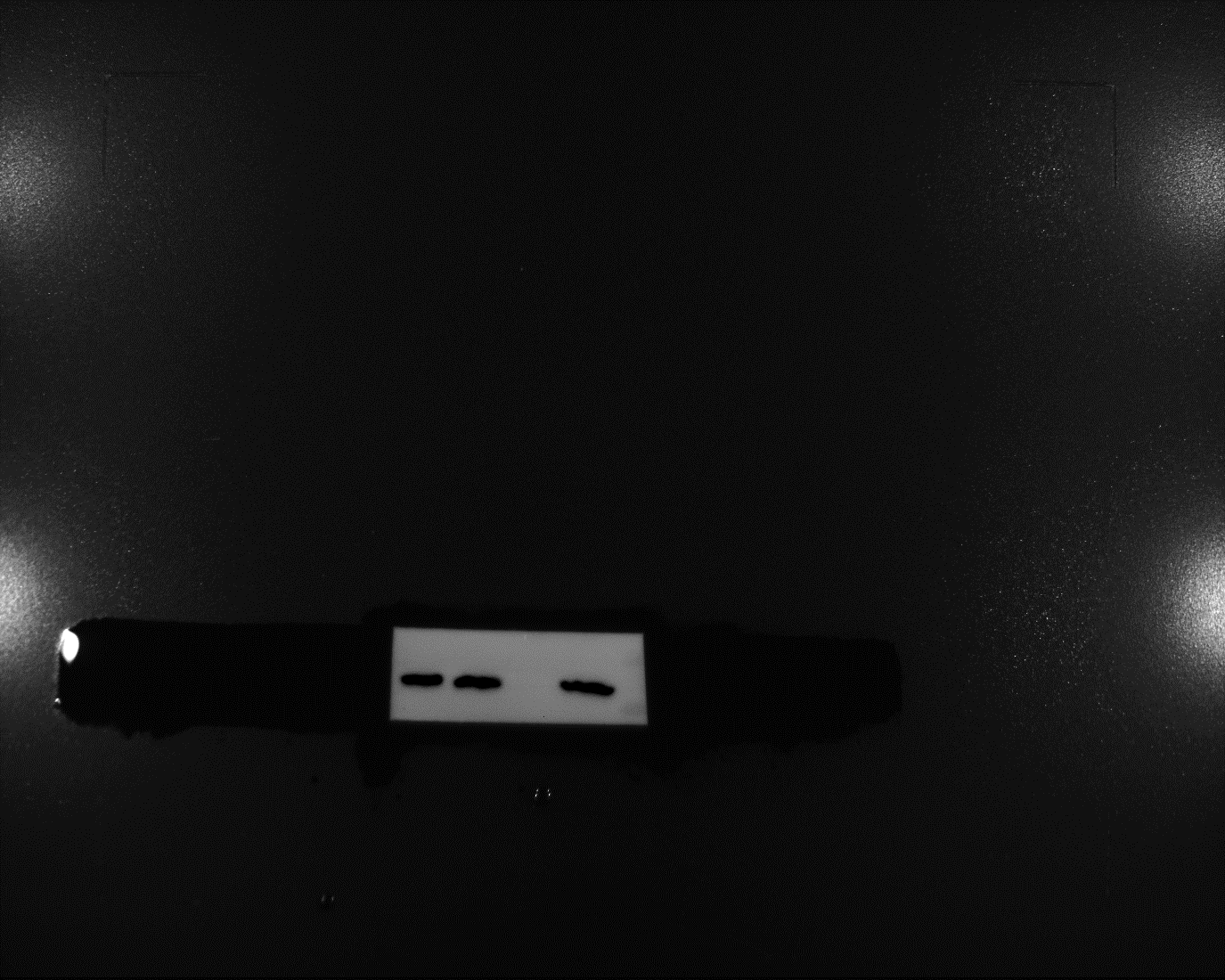


HDAC3^loxp^ Foxa1 AR


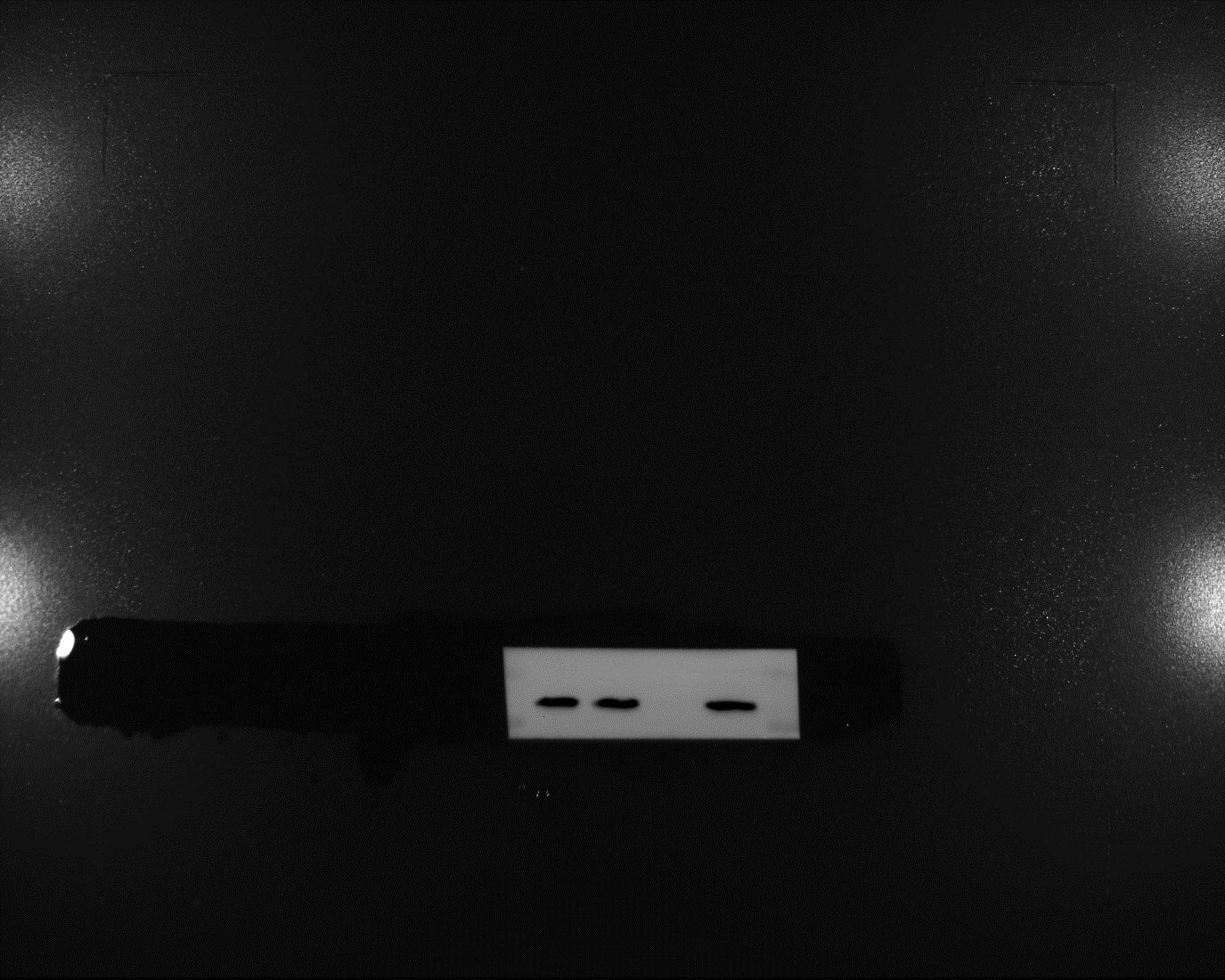


HDAC3^loxp^ Foxa2 AR


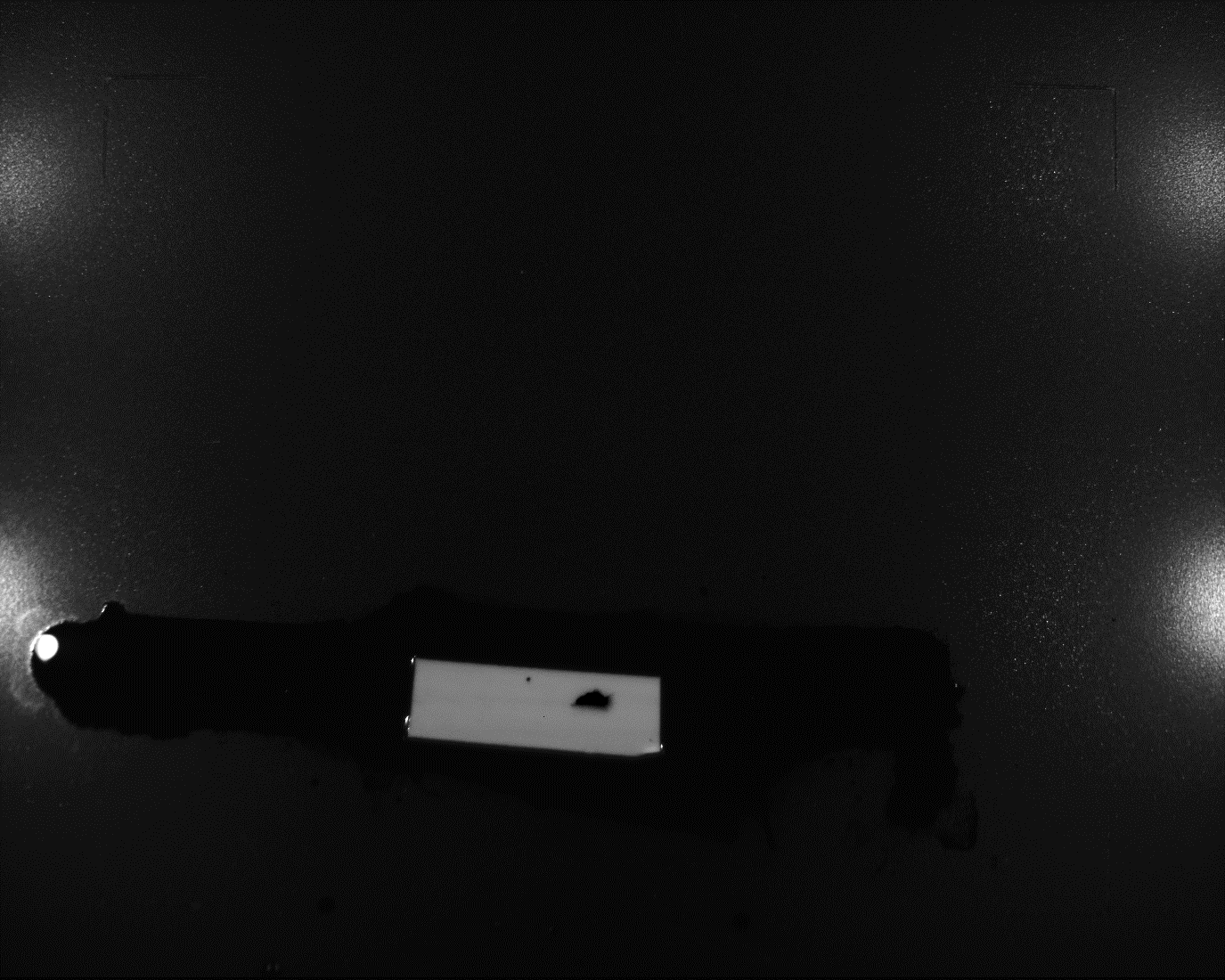


HDAC3^LCKO^ AR Foxa1


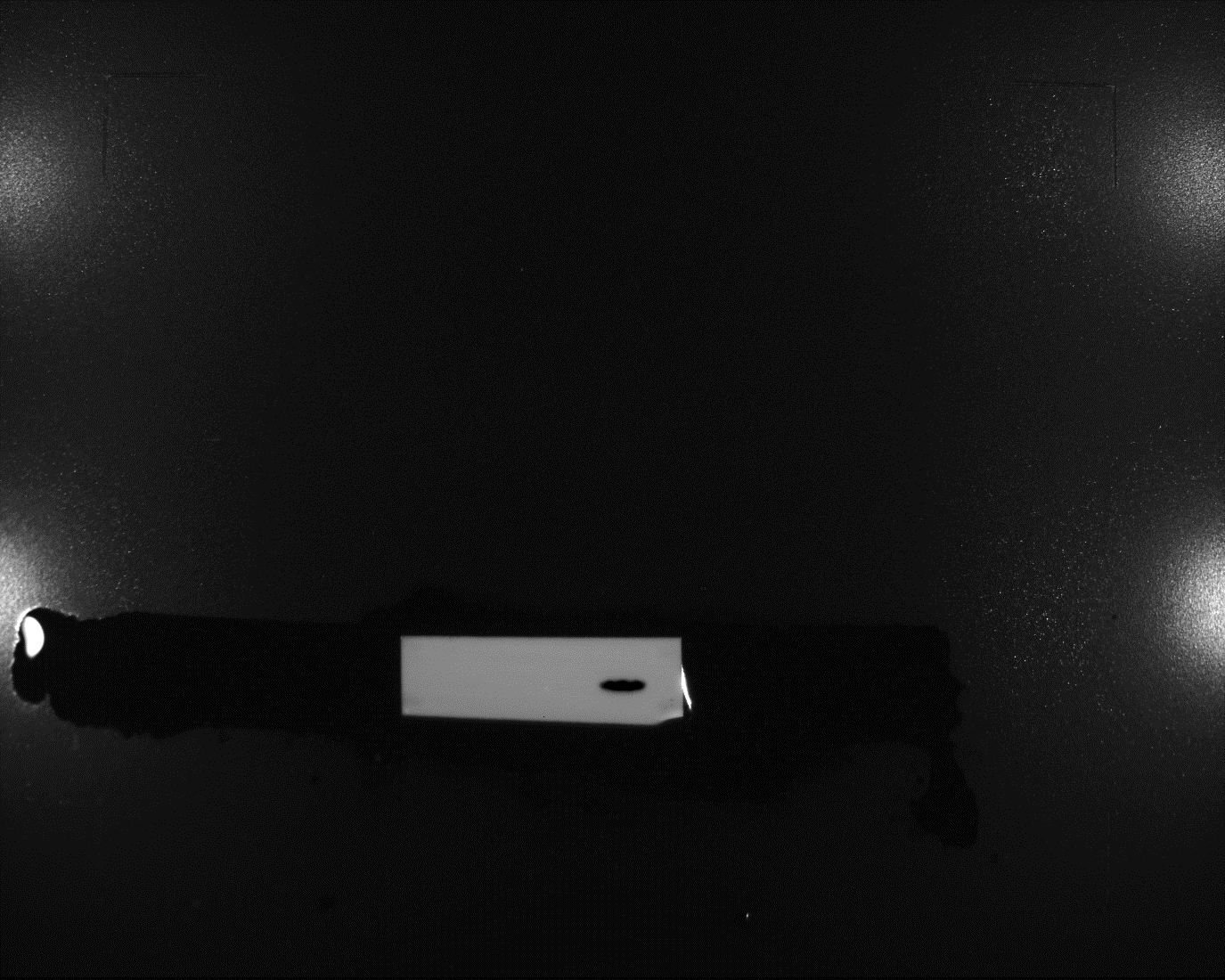


HDAC3^LCKO^ AR Foxa2


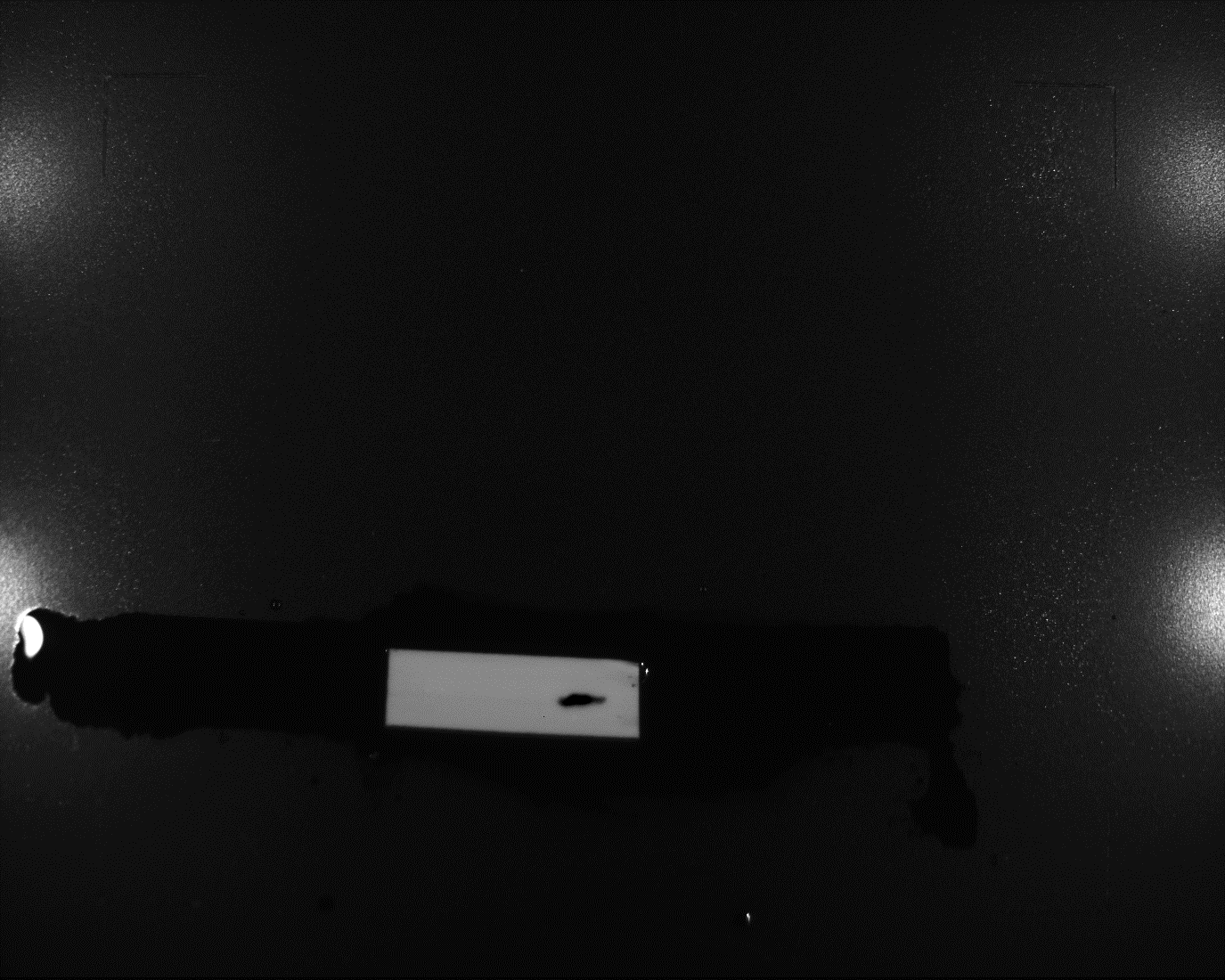


HDAC3^LCKO^ Foxa1 AR


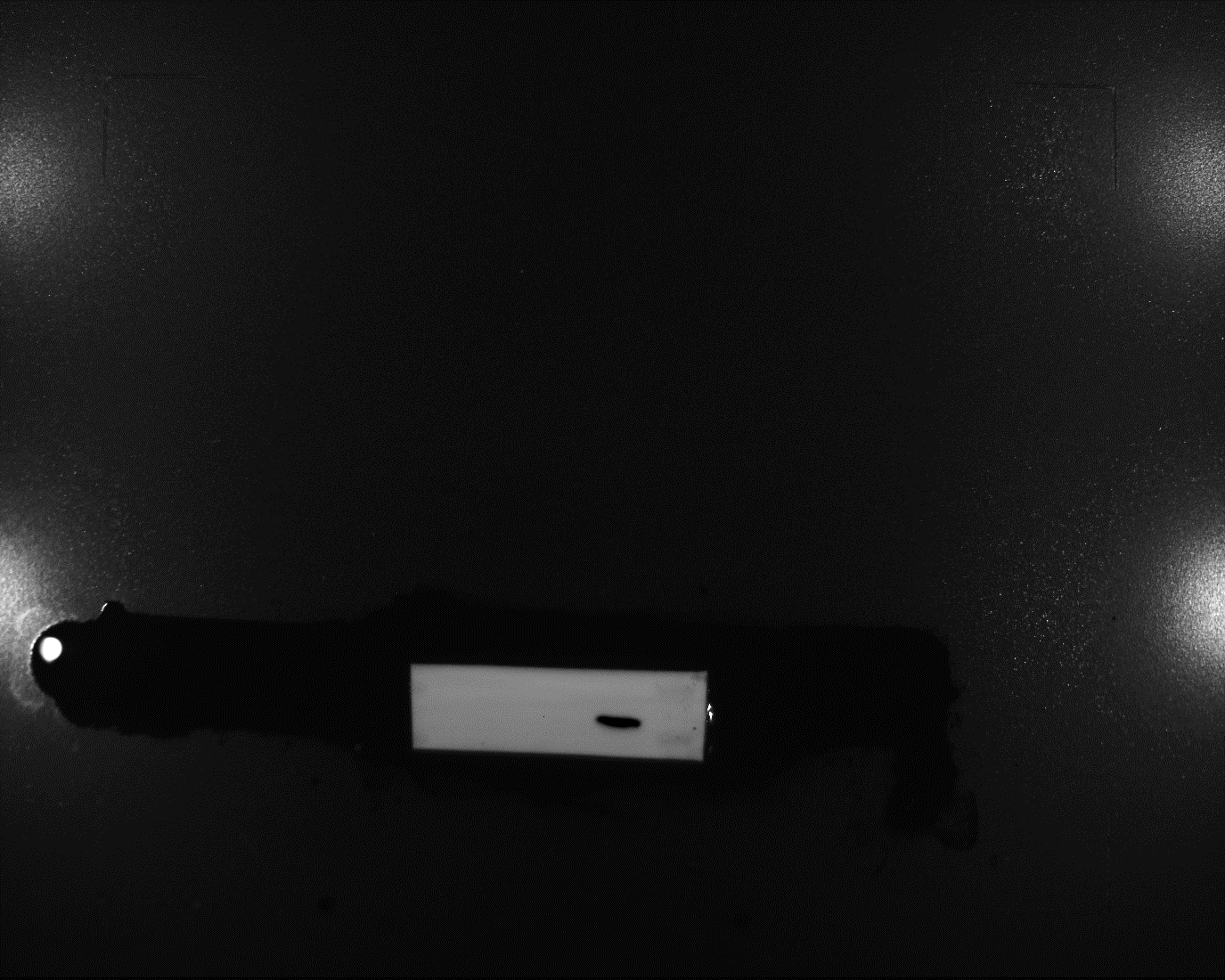


HDAC3^LCKO^ Foxa2 AR

Figure S1


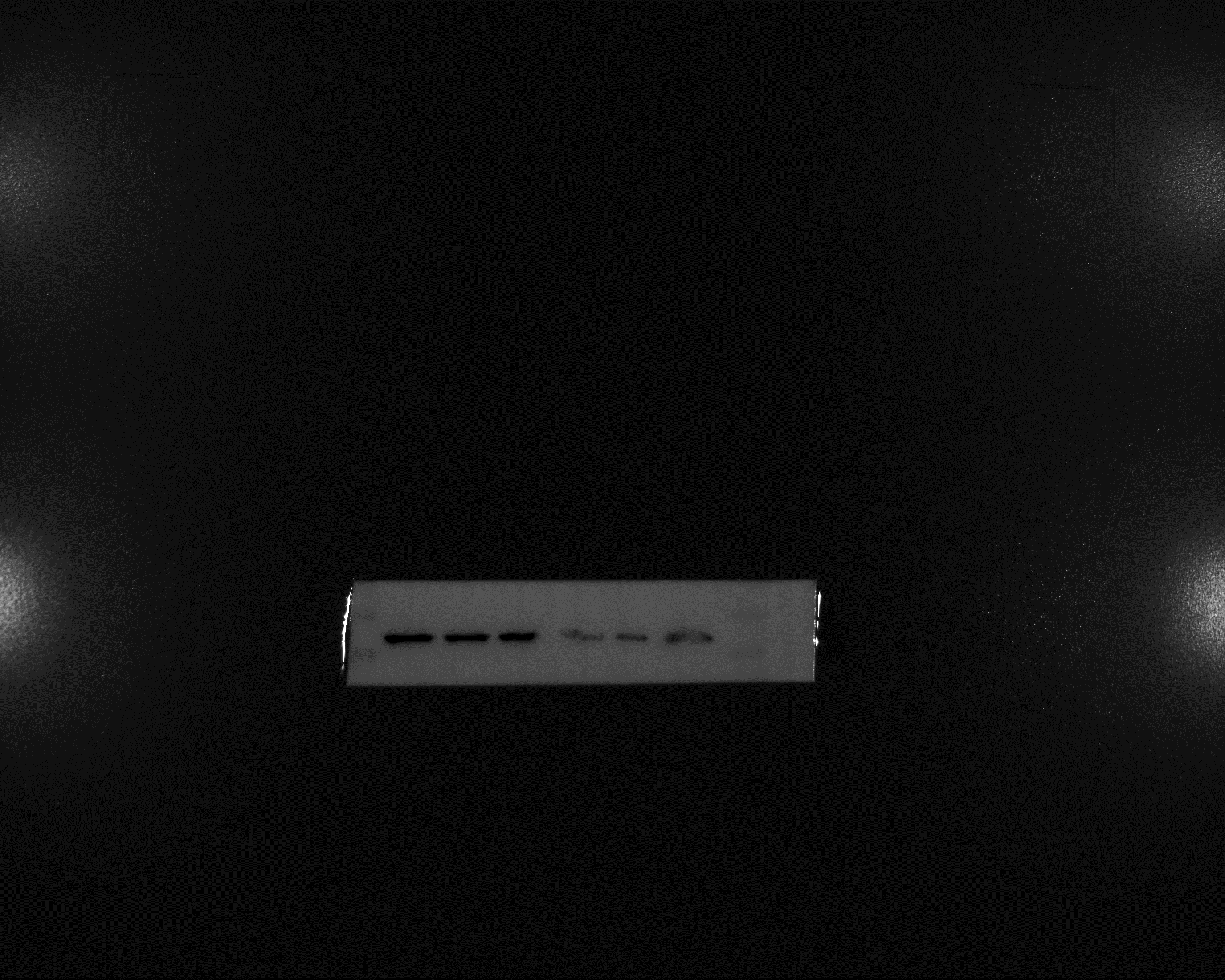


PPM1L


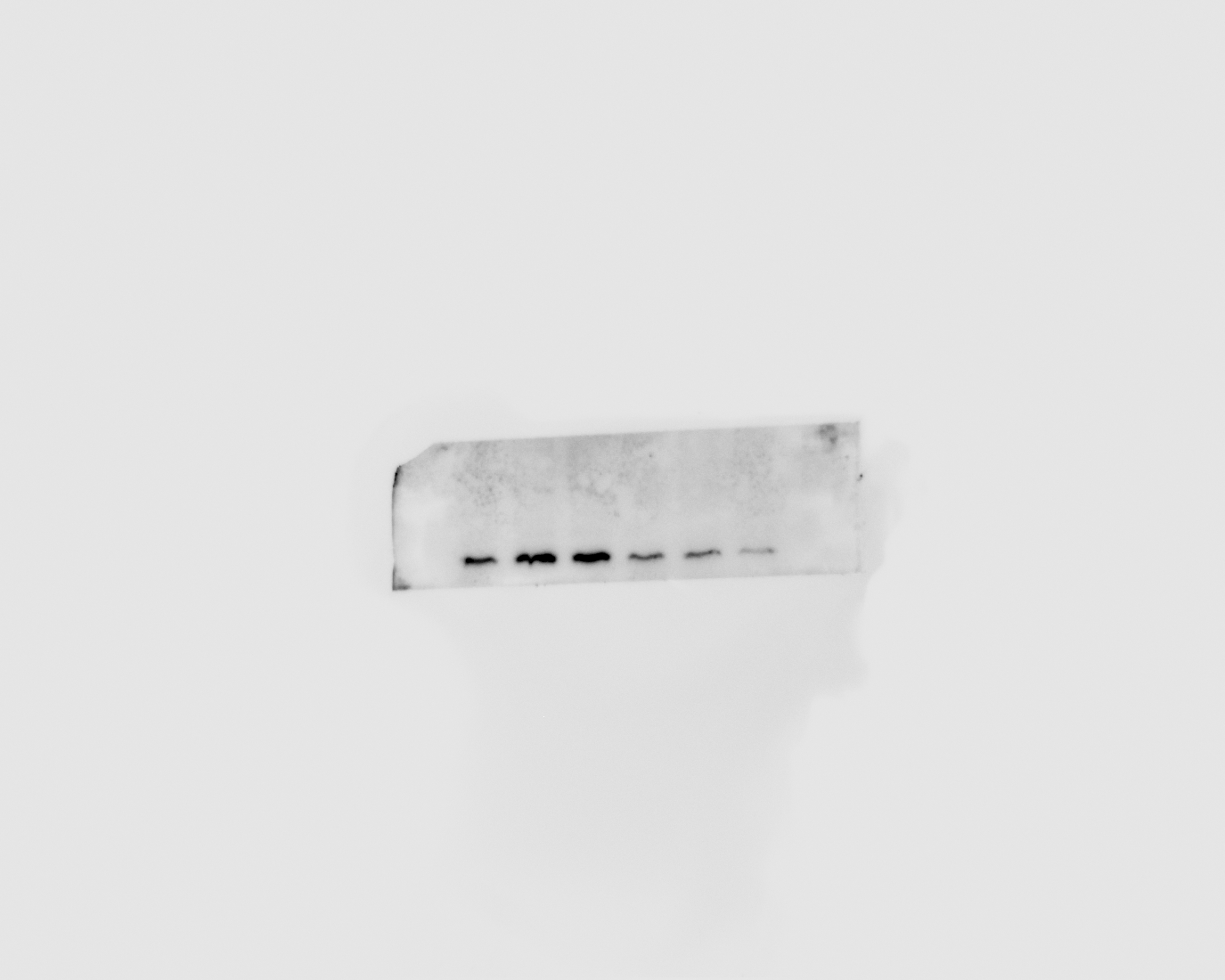


FGL1


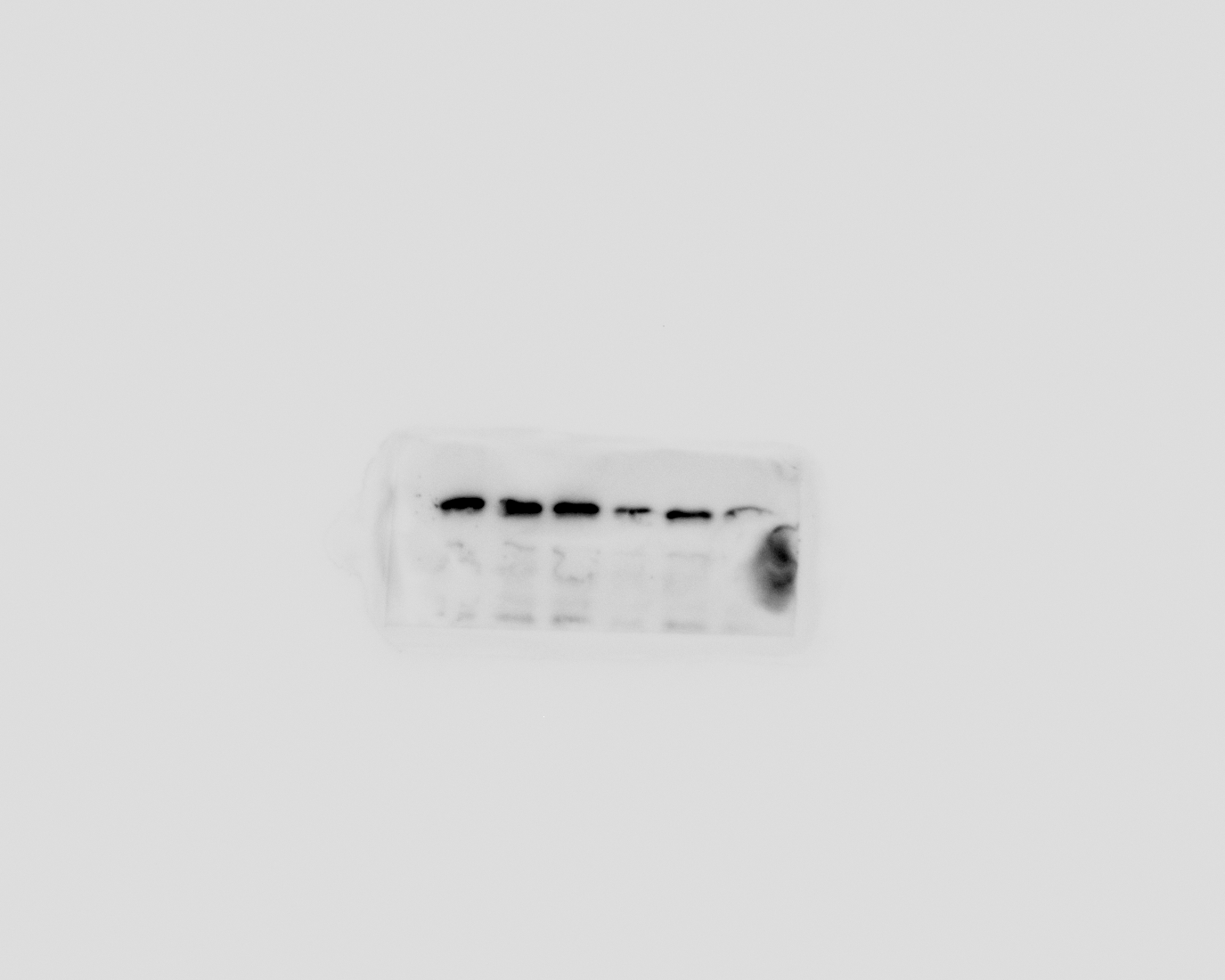


BTG1


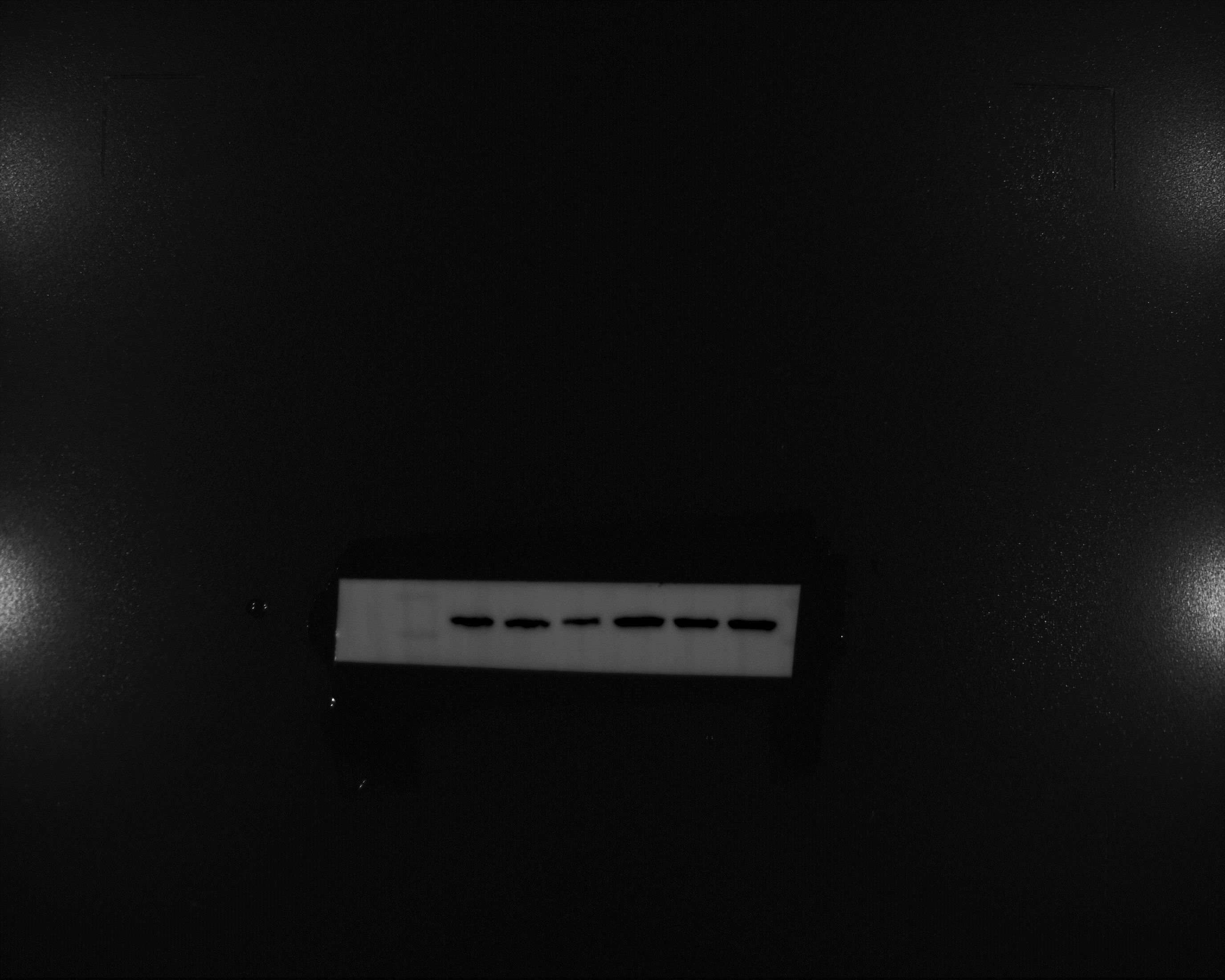


ABCC4


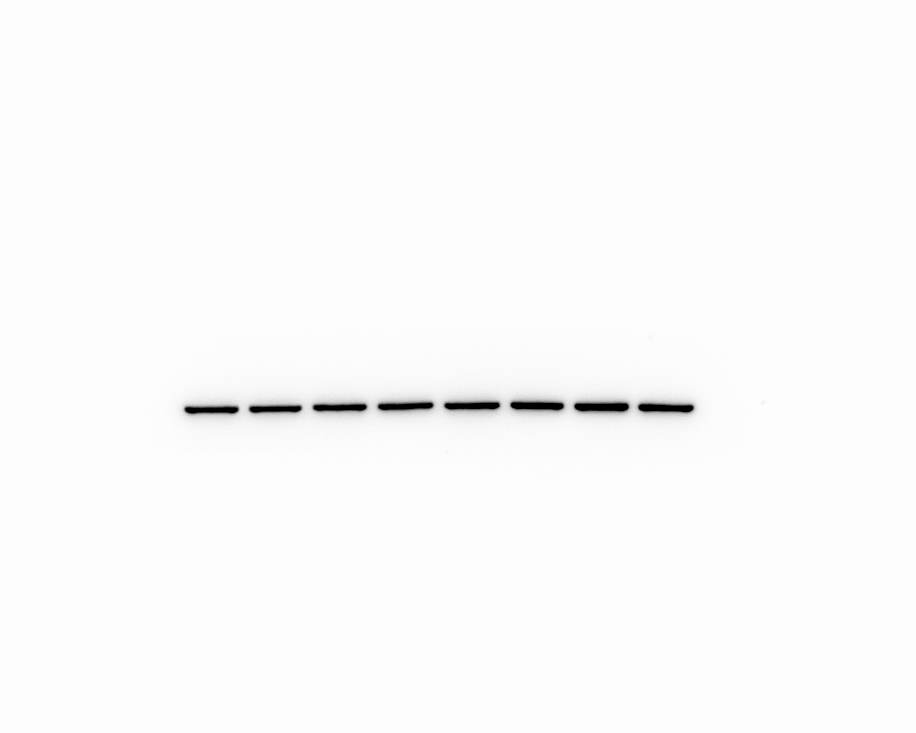


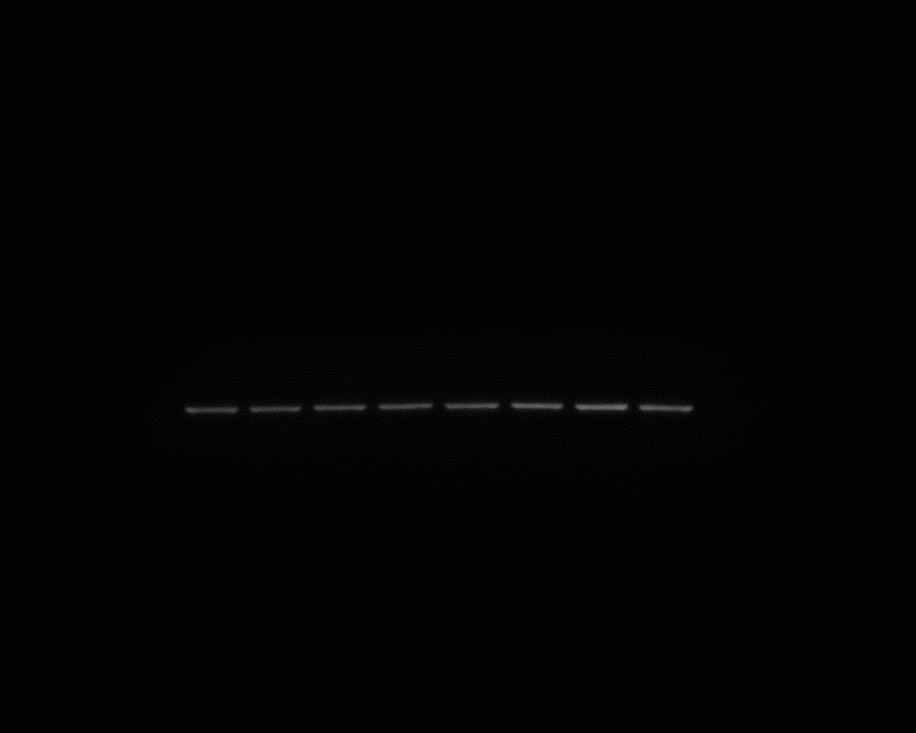


GAPDH
